# Expression analysis of candidate genes for fatty acid composition in adipose tissue and identification of regulatory regions

**DOI:** 10.1038/s41598-018-20473-3

**Published:** 2018-02-01

**Authors:** Manuel Revilla, Anna Puig-Oliveras, Daniel Crespo-Piazuelo, Lourdes Criado-Mesas, Anna Castelló, Ana I. Fernández, Maria Ballester, Josep M. Folch

**Affiliations:** 1 Animal Genomics Department, Centre for Research in Agricultural Genomics (CRAG), CSIC-IRTA-UAB-UB, Campus UAB, 08193 Bellaterra, Spain; 2 Departament de Ciència Animal i dels Aliments, Facultat de Veterinària, Universitat Autònoma de Barcelona (UAB), 08193 Bellaterra, Spain; 3 Departamento de Genética Animal, Instituto Nacional de Investigación y Tecnología Agraria y Alimentaria (INIA), 28040 Madrid, Spain; 4 Departament de Genètica i Millora Animal, Institut de Recerca i Tecnologia Agroalimentàries (IRTA), Torre Marimon, 08140 Caldes de Montbui, Spain

## Abstract

The aim of this work was to study the genetic basis of the backfat expression of lipid-related genes associated with meat quality traits in pigs. We performed a genome-wide association study with the backfat gene expression measured in 44 genes by qPCR and the *PorcineSNP60 BeadChip* genotypes in 115 Iberian x Landrace backcross animals. A total of 193 expression-associated SNPs located in 19 chromosomal regions were associated with expression levels of *ACSM5*, *ELOVL6*, *FABP4*, *FADS2*, and *SLC27A4* genes. Three expression quantitative trail loci (eQTLs) corresponding to *ACSM5*, *FABP4*, and *FADS2* were classified as *cis*-acting eQTLs, whereas the remaining 16 eQTLs have *trans*-regulatory effects. Remarkably, a SNP in the *ACSM5* promoter region and a SNP in the 3′UTR region of *FABP4* were the most associated polymorphisms with the *ACSM5* and *FABP4* expression levels, respectively. Moreover, relevant lipid-related genes mapped in the *trans*-eQTLs regions associated with the *ACSM5*, *FABP4*, *FADS2*, and *SLC27A4* genes. Interestingly, a *trans*-eQTL hotspot on SSC13 regulating the gene expression of *ELOVL6*, *ELOLV5*, and *SCD*, three important genes implicated in the elongation and desaturation of fatty acids, was identified. These findings provide new data to further understand the functional regulatory mechanisms implicated in the variation of fatty acid composition in pigs.

## Introduction

Pork meat is an appreciated, all-purpose lean meat, and represents one of the main sources of animal meat for humans^1^. Fat and fatty acids (FAs), are fundamental to various aspects of meat quality and play a crucial role to meat nutritional value, both in adipose tissue (backfat, BF) and muscle (intramuscular fat^2^). It is demonstrated that FA composition is dependent on physiological status, nutrition conditions^2,3^, and genetic factors^4^.

In the last few years, genome-wide association studies (GWAS) have been performed in attempts to uncover the genetic basis of FA composition traits. Studies of our group and others have benefited from this approach and have identified genomic regions significantly associated with intramuscular fatty acid (IMFA) composition by using different experimental and commercial populations^5–9^. However, in most cases, the GWAS approach only allows the detection of genetic variants that explain a modest proportion of the total heritability of the analyzed traits^10^. In addition, the way from the genomic statistical association to the identification of true causal genetic variants is plagued of difficulties. Hence, it has become evident the necessity to integrate new approaches to better understand the biological significance of GWAS findings. Recently, the association between genetic variants and gene expression levels has been described and used to identify expression quantitative trait *loci* (eQTLs). The eQTLs regulating the transcript abundance of the mRNAs can be identified systematically using high-throughput technologies and have recently been proposed as a good strategy to deepen the study of the genetic architecture of complex traits^11,12^.

Liver, skeletal muscle and adipose tissue are three of the most important tissues involved in FA metabolism^13^. Adipose tissue, one of the main energy reserves in animals, is composed of adipocytes embedded in a matrix of connective tissue with a highly developed vascular system. The adipocytes are dynamic cells that play a relevant role in energy balance and overall body homeostasis. Their main metabolic functions are to accumulate lipids, by synthesis of triacylglycerols, and lipid mobilization, through hydrolysis of triacylglycerols^14^.

Previous studies in our group have identified genomic regions, candidate regulators and regulatory polymorphisms in the liver and muscle tissues of individuals of an Iberian x Landrace backcross population (BC1_LD^15,16^). Furthermore, a RNA-Seq transcriptome study of the adipose tissue of two groups of pigs with phenotypically extreme IMFA composition in the BC1_LD animals identified metabolic pathways differentially modulated between groups controlling lipid and FA metabolism^17^. Taking into account the relevant role of adipose tissue in the regulation of lipid metabolism, the goals of the present work were (1) to study the expression of candidate genes related with lipid metabolism in the adipose tissue of BC1_LD animals and (2) to analyze the chromosomal regions significantly associated with the gene expression levels to characterize the regulatory mechanisms influencing gene expression phenotypes. With these results we aim to increase our knowledge of the regulation of key genes determining FA composition in pigs in adipose tissue.

## Results and Discussion

### Selection of genes related with lipid metabolism in the adipose tissue

Using the information generated in previous studies of our group, strong candidate genes affecting FA composition of BF and intramuscular fat in the BC1_LD generation were identified by GWAS, RNA-Seq and co-association network approaches^5,17–21^ (Supplementary Table S1). A total of 44 candidate genes related with lipid metabolism were selected to study their expression pattern in BF. Fourteen of them (*ARNT*, *CYP2U1*, *EGF, ELOVL6*, *FABP4*, *FABP5*, *FADS1*, *FADS2*, *FADS3, NFKB1*, *PLA2G12A*, *PLCB2*, *PLPP1*, and *USF1*) are functional and positional candidate genes related with lipid metabolism which were identified in GWAS analyses for BF and IMFA composition in the BC1_LD animals^5,21^. We also included two candidate genes differentially-expressed (*ELOVL6* and *SCD*) in a RNA-Seq analysis of the adipose tissue of two phenotypically extreme groups of animals for IMFA composition in the BC1_LD cross^17^. Lipid metabolism genes identified in gene co-association networks for FA composition (*ACSM5*, *ANK2*, *ARNT*, *FABP4*, *FABP5*, *MGLL*, and *PPARG*)^19^ were also included; two of which, *ARNT* and *PPARG*, were also identified in gene co-association networks for fatness and growth traits^20^. In addition, in order to complete the set of genes, we included genes which have been described in the literature to play different roles in lipid metabolism such as transporters (*RBP4*, *SCAP*, *SLC27A1*, and *SLC27A4*), enzymes (*AGPAT2*, *CPT1A*, *CROT*, *DGAT1*, *DGAT2*, *ELOVL5*, *LIPC*, *LPIN1*, *PEX2*, and *PNPLA2*) and transcriptional factors, cofactors or nuclear receptors (*CD36, ESRRA, MLXIPL*, *NR1H3*, *POU2F1*, *PPARA*, *PPARD*, *PPARGC1A*, *RXRG*, and *SREBF1*). Finally, we added the *ADIPOQ* gene, which encodes an important adipokine of white fat tissue, exerting multiple biological processes on carbohydrate and lipid metabolism (review in Shehzad *et al*.^22^). Although this gene is not annotated in the current *Sscrofa10.2* assembly, it has been mapped to the SSC13q36-41 interval^23^.

Four reference genes (*ACTB, B2M, HPRT*, and *TBP*) were also selected as reference controls.

### Expression genome-wide association analysis

In the present study, the adipose tissue expression of 48 genes (44 target and four reference genes) was measured by quantitative real-time PCR (qPCR) in 115 BC1_LD animals. For the *PPARGC1A* gene, a poor PCR efficiency was obtained and it was discarded for further analysis. Expression GWAS (eGWAS) were performed with the gene expression values of the 43 remaining target genes and the genotypes of 40,460 SNPs of the *PorcineSNP60 BeadChip* (Illumina) that passed the quality control (see Methods section).

**Figure 1:**
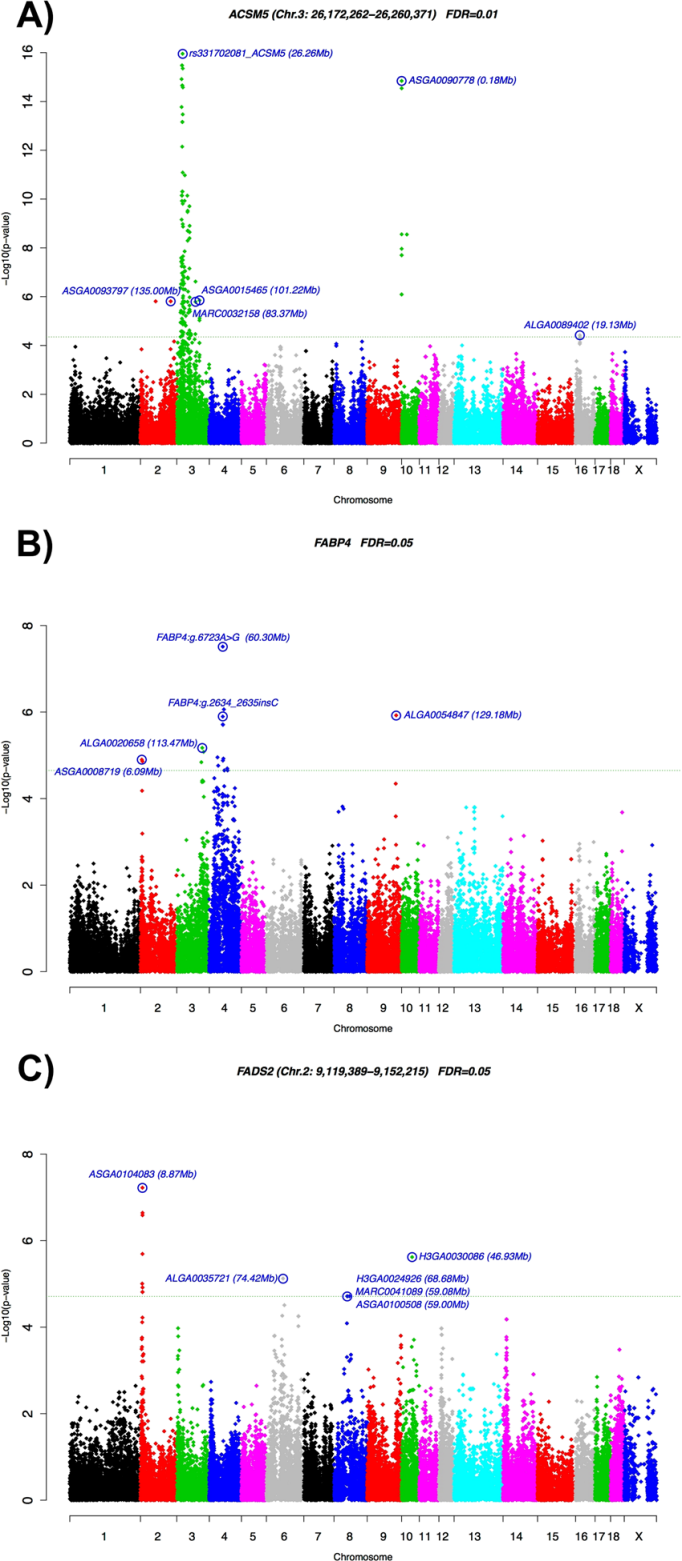
GWAS plot of *ACSM5*, *FABP4*, and *FADS2* gene expression in adipose tissue. The X-axis represents chromosome positions in Mb relative to *Sscrofa10.2* assembly of the pig genome and the Y-axis shows the –log10 (*p*-value). Horizontal dashed lines indicate the genome-wide significance level. Plot of eGWAS for (**A**) *ACSM5*, (**B**) *FABP4*, and (**C**) *FADS2* gene expression in backfat.

At whole genome level (*q*-value ≤ 0.05), significant association signals in five of the analyzed genes were detected (Table 1, Fig. 1, Supplementary Fig. S1): *ACSM5, ELOVL6, FABP4, FADS2*, and *SLC27A4*. The *ACSM5, FABP4, FADS2*, and *SLC27A4* genes presented more than one associated eQTL (Table 1). Three out of 19 eQTLs were identified as *cis*-acting for the *ACSM5, FABP4*, and *FADS2* gene expression (Fig. 1), suggesting the presence of proximal polymorphisms regulating the expression of these genes. These results showed a difference in the ratio of *cis*- and *trans*-eQTLs. In general, studies performed in animals have identified regulatory *trans*-eQTLs in a higher ratio than those performed in humans^12,24^. Accordingly, previously eGWAS analyses performed in muscle^15^ and liver^16^ of BC1_LD animals identified a prevalence of porcine *trans*-eQTLs compared wih *cis*-eQTLs.

**Table 1:** Significant eQTL identified.

Interval	Chr	Position Mb Start-End^a,b^	Size (Mb)	SNP Start	SNP End	No. SNPs^c^	Associated Gene	Type of eQTL
I1	2	134.99		ASGA0093797		1	*ACSM5*	*trans*
I2	3	16.47–63.25	46.78	ASGA0089930	ALGA0111911	133	*ACSM5*	*cis/trans*
I3	3	83.37		MARC0032158		1	*ACSM5*	*trans*
I4	3	100.91–101.46	0.55	ALGA0020206	ASGA0098441	3	*ACSM5*	*trans*
I5	10	0.05–0.20	0.15	H3GA0055101	ASGA0095156	8	*ACSM5*	*trans*
I6	16	19.13		ALGA0089402		1	*ACSM5*	*trans*
I7	13	6.89		ASGA0055780		1	*ELOVL6*	*trans*
I8	2	6.10–9.00	2.90	ASGA0008719	MARC0018949	2	*FABP4*	*trans*
I9	3	109.40–119.74	10.34	ASGA0015643	ASGA0016181	3	*FABP4*	*trans*
I10	4	36.73		ALGA0024527		1	*FABP4*	*trans*
I11	4	60.57–65.25	4.68	ALGA0025158	ALGA0025337	5	*FABP4*	*cis* (*FABP5*)^d^
I12	9	129.18		ALGA0054847		1	*FABP4*	*trans*
I13	2	7.85–9.22	1.37	ASGA0008845	ASGA0008874	8	*FADS2*	*cis*
I14	6	74.42		ALGA0035721		1	*FADS2*	*trans*
I15	8	59.00–68.68	9.68	ASGA0100508	H3GA0024926	3	*FADS2*	*trans*
I16	10	46.93		H3GA0030086		1	*FADS2*	*trans*
I17	9	20.18–20.20	0.02	MARC0034587	ASGA0041925	2	*SLC27A4*	*trans*
I18	14	88.90–92.43	3.53	ASGA0064787	ALGA0079407	16	*SLC27A4*	*trans*
I19	15	137.05–137.37	0.32	ASGA0070790	MARC0050960	2	*SLC27A4*	*trans*

^a^Chromosomal location is given according to *Sscrofa10.2* assembly coordinates.

^b^Gene annotation was performed including one additional Mb at the start and at the end of the *trans* eQTL regions. For the *cis* eQTLs only the analyzed gene was considered.

^c^Number of significant SNPs within the eQTL interval.

^d^The known mapped *FABP5* gene (SSC4: 60.31 Mb) was used in order to define *cis/trans* eQTLs for the unmapped *FABP4* gene.

The eGWAS identified 193 expression-associated SNPs located in 19 chromosomal regions on pig chromosomes SSC2-SSC4, SSC6, SSC8-SSC10 and SSC13-SSC16 (Table 1).

From the associated SNPs, and according to the Variant Effect Predictor (VEP) of Ensembl (*Sscrofa10.2* annotation release 84), 49.2% (95 SNPs) were located in intergenic regions. The remaining 50.8% (98 SNPs) mapped within a total of 68 genes: 66 in introns, 11 in 5′ upstream and 16 in 3′ downstream gene regions, three in the 3′UTR region, and two in the coding region of a gene determining synonymous mutations (Supplementary Table S2). A total of 86 SNPs (44.6%) were located inside the *cis*-acting eQTLs, whereas 107 SNPs (55.4%) were in *trans-*eQTLs (Supplementary Table S2).

In the following sections, the SNPs and genes mapped in the eQTL regions associated with the expression phenotypes of *ACSM5, ELOVL6, FABP4*, *FADS2*, and *SLC27A4* are discussed in detail. The selection of genes in the eQTLs was performed by: (1) extracting the annotated genes from the eQTL intervals including an additional Mb at each end, (2) selecting genes with a lipid-related function and (3) expressed in adipose tissue. Table 2 summarizes all the relevant lipid-related genes mapped in the *trans*-eQTL regions.

**Table 2:** Candidate genes annotated in *trans*-eQTLs related with lipid metabolism functions.

Gene	Chr	Interval	Candidate gene within eQTL
*ACSM5*	2	I1	*ALDH7A1, MARCH3*
	3	I3	*MDH1*
	3	I4	*PIGF, PRKCE*
	10	I5	*COG7*, *GGA2, NDUFAB1*
*FABP4*	2	I8	*BSCL2, DAGLA, EHD1, FADS1, FADS2, FADS3, LGALS12, PLA2G16, SF1, TM7SF2*
	3	I9	*CYP1B1, EHD3, EPT1, GALNT14, GCKR, HADHA, HADHB, LCLAT1, PLB1, PPP1CB, RBKS, SNX17, SPAST*
	9	I12	*RFWD2*
*FADS2*	6	I14	*CDC42*, *ECE1*, *FUCA1*, *GALE*, *HMGCL*, *KDM1A*
	8	I15	*IGFBP7*
	10	I16	*CUBN*
*SLC27A4*	9	I17	*PRCP*
	15	I19	*FARSB*, *MOGAT1*

#### The *ACSM5* gene

The most significant *cis*-SNPs (*p*-value = 1.11 × 10^−17^) for the Acyl-CoA Synthetase Medium-Chain Family Member 5 (*ACSM5*) gene expression were located between 23.44 Mb and 27.94 Mb on SSC3, covering a total of 25 SNPs (Supplementary Table S2), in a region where these SNPs have a high linkage disequilibrium (LD) (r^2^ = 0.42–0.99). Recently, the polymorphism *ACSM5:g.26260422* *G*>*A* (rs331702081), located at the proximal promoter region of the *ACSM5* gene, has been described as the most associated with the expression of *ACSM5* in *Longissimus dorsi* muscle of BC1_LD animals^15^. Hence, the *ACSM5:g.26260422* *G*>*A* SNP was genotyped in the 115 BC1_LD animals and included in the eGWAS study, showing that is one of the SNPs located in the 23.44–27.94 Mb block with the lowest *p*-value (*p*-value = 1.11 × 10^−17^; Fig. 1A). Consequently, this SNP may be a strong candidate to explain the mRNA variation of the *ACSM5* gene in BF and muscle. Even so, the correlation value between the *ACSM5* gene expression in BF and muscle is of r = 0.60 (*p*-value = 3.0 × 10^−12^), suggesting that other factors than the SSC3 *cis*-eQTL may be differentially regulating the expression of *ACSM5* in both tissues.

The effect of *ACSM5:g.26260422* *G*>*A* SNP on the binding of transcription factors (TFs) was determined by the LASAGNA-Search version 2.0 software^25^. The aryl hydrocarbon receptor nuclear translocator (*ARNT*) gene (TF ID = M00236 and M00539) and the signal transducer and activator of transcription 6 (*STAT6*) gene (TF ID = M00500) were identified to bind only when the *A* allele is present. *ARNT* gene plays an important role in the regulation of hepatic lipogenesis and gluconeogenesis^26,27^, and was identified as one of the most central genes in a liver co-expression network analysis of IMFA composition in pigs^19^. *STAT6* gene has been described to interact with the peroxisome proliferator activated receptor gamma gene (*PPARG*), and the cooperative binding of the two genes led to an increase response of *PPARG*^28^. The importance of *PPARG* gene lies on its regulation of adipocyte differentiation and glucose homeostasis, and it was identified as a major regulator for growth and fatness related traits in a co-association network in muscle of BC1_LD individuals^20^. Different transcription binding sites for *PPARG* in the *ACSM5* proximal promoter region were also predicted by LASAGNA (Supplementary Table S3).

Five chromosomal regions (Table 1) were also associated in *trans* with the *ACSM5* gene expression. Likewise, two of these regions on SSC3 (100.91–101.46 Mb) and SSC10 (0.05–0.20 Mb) have been recently associated with the mRNA expression of the *ACSM5* gene in the *Longissimus dorsi* muscle of BC1_LD animals (SSC3: 100.35 Mb; SSC10: 0.17 Mb)^15^. These results, together with the *cis*-eQTL identification for the *ACSM5* expression, further confirm the existence of common regulatory mechanisms implicated in the expression of *ACSM5* in BF and muscle. No strong candidate genes exerting a known lipid metabolism function were detected in the *trans*-eQTL identified on SSC16 for *ACSM5*. Conversely, a list of lipid-related genes identified on SSC2, SSC3 and SSC10 *ACSM5*-*trans* associated regions is showed in Table 2.

#### The *ELOVL6* gene

In the present study, previously described *ELOVL6* polymorphisms (three in the promoter region, one in exon 4 and two in the 3′UTR region)^18,29^ were genotyped in the 115 BC1_LD animals and incorporated in the eGWAS. The obtained results were consistent with our previous studies on SSC8^18^, showing a chromosome level significant region associated with *ELOVL6* mRNA expression in BF (Supplementary Fig. S2). The most significant peak was for two SNPs with the same *p*-value (ALGA0049135 and ALGA0049139; *p*-value = 4.60 × 10^−5^; â = 0.563), located in an intron of *ANK2*, at 2.4 Mb to the *ELOVL6* gene. In contrast the *ELOVL6:c.-533C*>*T* and *ELOVL6:c.-394G*>*A* polymorphisms, which were in full LD and also showed high association (*p*-value = 6.42 × 10^−04^; â = 0.460), did not achieve statistical significance after multiple testing corrections (*q*-value ≤ 0.05; Supplementary Fig. S2). This observed discrepancy could be due to the different animals used between the two studies (94 samples overlapping between the two studies) and to the subtle differences observed between the two methods used for mRNA quantification (Pearson correlation coefficient, r = 0.91).

Interestingly, *ANK2* is one of the most central genes in an adipose co-expression network related with IMFA composition in the BC1_LD^19^. In a recent study, knockin mice expressing *ANK2* genes with human nonsynonymous mutations showed altered glucose homeostasis contributing to increased adiposity. This phenotype was caused by the reduction in ANK2 protein levels which produce an elevation of cell surface GLUT4 and increased glucose uptake in skeletal muscle and fat^30^. Thus, we cannot discard an association of this gene with the mRNA expression levels of *ELOVL6*. Further analyses are necessary to corroborate this hypothesis.

Regarding the *trans*-associated regions with the BF mRNA *ELOVL6* expression, Corominas *et al*.^18^ did not identify any region at whole genome level (*q*-value ≤ 0.05), in contrast with the *trans*-eQTL on SSC13 identified in the present study. No genes related to lipid metabolism function were identified in the SSC13 *trans*-eQTL. However, we have to highlight that the same region on SSC13 was associated at chromosome level with the expression of the *ELOVL5* and *SCD* genes. *ELOVLs* and *SCD* genes are implicated in the elongation and desaturation of FAs in the endoplasmic reticulum membrane. These metabolic functions are essential to the maintenance of lipid homeostasis (reviewed in Guillou *et al*.^31^). Moreover, it has been reported that the expression of these genes are primarily regulated at transcriptional level (reviewed in Guillou *et al*.^31^). In this study, highly significant Pearson’s correlations (*p*-value = 2.22 × 10^−16^) among the mRNA expression of these genes were obtained (r_*ELOVL5-ELOVL6*_ = 0.90; r_*ELOVL5-SCD*_ = 0.84; r_*ELOVL6-SCD*_ = 0.86), supporting the involvement of common elements regulating their mRNA expression. In addition, we found high correlations with the genes coding for the TFs SREBF1 and PPARs (r_*ELOVL5-PPARG*_ = 0.75, *p*-value = 2.22 × 10^−16^; r_*ELOVL5-SREBF1*_ = 0.74, *p*-value = 2.22 × 10^−16^; r_*ELOVL5-PPARA*_ = 0.65, *p*-value = 1.02 × 10^−14^; r_*ELOVL6-PPARG*_ = 0.68, *p*-value = 2.22 × 10^−16^; r_*ELOVL6-SREBF1*_ = 0.70, *p*-value = 2.22 × 10^−16^; r_*ELOVL6-PPARA*_ = 0.69, *p*-value = 2.22 × 10^−16^; r_*SCD-PPARG*_ = 0.59, *p*-value = 1.01 × 10^−11^; r_*SCD-SREBF1*_ = 0.63, *p*-value = 8.48 × 10^−14^; r_*SCD-PPARA*_ = 0.51, *p*-value = 1.03 × 10^−8^) which contribute to the regulation of these genes^17,31,32^. High correlation values were also found between the mRNA expression of *DGAT1* and *DAGT2* genes with *ELOVLs* and *SCD* genes (r_*DGAT1-ELOVL5*_ = 0.72, *p*-value = 2.22 × 10^−16^; r_*DGAT1-ELOVL6*_ = 0.70, *p*-value = 2.22 × 10^−16^; r_*DGAT1-SCD*_ = 0.59, *p*-value = 3.89 × 10^−12^; r_*DGAT2-ELOVL5*_ = 0.81, *p*-value = 2.22 × 10^−16^; r_*DGAT2-ELOVL6*_ = 0.74, *p*-value = 2.22 × 10^−16^; r_*DGAT2-SCD*_ = 0.76, *p*-value = 2.22 × 10^−16^). This result agrees with the interrelated function of these genes which are implicated in lipogenesis (*ELOVLs* and *SCD*) and triglyceride (TG) synthesis (*DGAT1* and *DGAT2*), the main function of adipose tissue^14^.

*ELOVL6* and *SCD* genes were over-expressed in the adipose tissue transcriptome (RNA-Seq) of BC1_LD animals with higher content of intramuscular monounsaturated fatty acids (MUFA) and saturated fatty acids (SFA), when compared with animals having more polyunsaturated fatty acids (PUFA)^17^ supporting the relevance of these genes in the determination of FA composition in the BC1_LD.

#### The *FABP4* gene

The Fatty Acid Binding Protein 4 (*FABP4*) gene is not annotated in the current *Sscrofa10.2* assembly. Nevertheless, radiation hybrid (RH) and linkage maps located *FABP4* gene close to *FABP5* on SSC4^33,34^, in agreement with the human comparative map. For this reason, we used the position of the annotated *FABP5* gene (SSC4: 60.31 Mb) in order to define *cis/trans* eQTLs for *FABP4* gene. Recently, an indel located in the intron 1 of *FABP4* (*FABP4:g.2634_2635insC*) was identified as the most associated polymorphism with the *FABP4* mRNA levels in BF^35^. In the present study, this indel, which was genotyped in the 115 BC1_LD animals and included in the eGWAS study, was one of the most significant associated polymorphisms (*p*-value = 1. 27 × 10^−6^; â = −0.219) (Fig. 1B), but the most significant *cis*-SNP (ALGA0025337; *p*-value = 8.67 × 10^−7^; â = 0.328) was located in an intergenic region (Supplementary Table S2).

Taking into account the highly polymorphic nature of the porcine *FABP4* gene^36^ and a possible regulatory role of miRNAs in the *FABP4* gene expression^37^, the 3′UTR of the *FABP4* gene was amplified and sequenced using 10 animals with extreme values of *FABP4* mRNA expression in BF. A polymorphism in the 3′UTR (*FABP4:g.6723* *A*>*G*) was identified and genotyped in the 115 BC1_LD animals. The association analysis with the *FABP4* mRNA expression values revealed that the SNP in the 3′UTR region has the lowest *p*-value (*p*-value = 3.07 × 10^−8^; â = −0.260) (Fig. 1B).

Interestingly, *FABP4:g.6723* *A*>*G* SNP was inside a putative miRNA binding site for the putative human miRNA hsa-miR-3182 and it is predicted to bind only when *FABP4:g.6723* *G* allele is present. However, we did not find the homologous *Sus scrofa* miR-3182 in the current assembly (*Sscrofa10.2*) of the pig genome. Although the 3′UTR SNP (*FABP4:g.6723* *A*>*G*) is a clear candidate to explain the differences of *FABP4* mRNA levels among animals, we cannot discard also a role of the indel (*FABP4:g.2634*_*2635insC*) in the *FABP4* gene regulation. Indeed, the indel polymorphism was predicted to be located in a target binding site for PPARG and NR4A2 TFs^35^. In our analysis the correlation between the mRNA expression of *PPARG* and *FABP4* was r = 0.51 (*p*-value = 9.27 × 10^−9^). Further functional analyses are needed to test the role of these polymorphisms in the *FABP4* gene regulation and besides in the determination of IMFA composition.

The eGWAS performed for the *FABP4* gene also revealed four *trans*-eQTLs on SSC2, SSC3, SSC4, and SSC9 (Table 1); where several genes involved in lipid metabolism were mapped (Table 2).

It is noteworthy to highlight some genes identified in the *trans*-eQTLs, such as the Berardinelli-Seip Congenital Lipodystrophy 2 (Seipin) (*BSCL2*) gene located on SSC2. Although the function of this gene is still being investigated, its relationship with adipogenesis, with the genesis of lipid droplets and the regulation of the metabolism of phospholipids and triacylglycerides has been established^38^. Liu *et al*.^39^ performed an adipose-specific Seipin knockout mice with FABP-mediated *BSCL2* deletion exhibiting decreased lipolysis in response to β-adrenergic receptors agonists *in vivo*. In this chromosomal region we also identified the Lectin Galactoside Binding Soluble 12 (*LGALS12*) gene, an intracellular galectin preferentially expressed in adipocytes which regulates lipolysis and whole-body energy metabolism^40^. Furthermore, *FABP4* and *LGALS12* genes were identified in the glycolysis/gluconeogenesis pathway in an expression analysis performed in human adipose-derived stem cells^41^. Finally, the Phospholipase A2 Group XVI (*PLA2G16*) gene, which encodes the most abundant PLA_2_ in the adipose tissue^42^, was also identified on SSC2. *PLA2G16* has been described an important regulator of the rate of adipose tissue lipolysis via the production of eicosanoid mediators^42^. On SSC3, we identified the Cytochrome P450 Family 1 Subfamily B Member 1 (*CYP1B1*) gene. Liu *et al*.^43^ have demonstrated that the reduction in body weight gain and white adipose tissue in *CYP1B1* deficient mice exhibited coordinated decreases in FA synthesis (regulated by *FABP4*, among others) when compared with wild type ones.

#### The *FADS2* gene

The most significant *cis*-SNP for *FADS2* gene expression (ASGA0104083; *p*-value = 5.98 × 10^−8^; â = 0.466) was located less than 0.23 Mb upstream of the *FADS2* gene (Fig. 1C). *FADS2* cluster together with *FADS1* and *FADS3* in a region on SSC2^44^. In liver, a high correlation between the mRNA expression levels of *FADS1* and *FADS2* has been observed (r_*FADS1-FADS2*_ = 0.92; *p*-value = 1.11 × 10^−17^) and a common *trans*-associated chromosomal region was identified on SSCX^16^. Hence, these results pointed to common regulatory mechanisms controlling the mRNA expression of both desaturases in liver^16^. Conversely, in BF the correlation between the mRNA expression levels of *FADS1* and *FADS2* was lower (r_*FADS1-FADS2*_ = 0.63; *p*-value = 8.26 × 10^−14^) and only the *cis*-acting element associated with the *FADS2* mRNA expression was identified. Altogether these results suggest that common and specific elements are regulating the expression of desaturases in BF.

Conversely, the low correlation observed between *FADS2* mRNA levels in BF and liver (r = 0.23; *p*-value = 1.91 × 10^−2^) suggests a different mechanism of regulation in both tissues. In addition, the *FADS2* gene was differentially-expressed between sexes depending on the tissue. Whereas in adipose tissue the *FADS2* gene showed a higher expression in males, in liver the expression was higher in females^16^. Similar results have also been shown in rats, where the mRNA expression of *FADS2* is higher in the liver of females than males^45^. To find polymorphisms which may modulate *FADS2* expression a search of polymorphisms was performed by the analysis of whole-genome sequence data from seven founders of the IBMAP population with IGV^46^. Three polymorphisms were identified at positions *g9118843C*>*T* (rs331050552), *g9118813G*>*A* (rs321384923), and *g9118721G*>*A* (rs336076510) according to the Ensembl ENSSSCG00000013072 sequence. The most proximal 5′ mutation (*g9118843C*>*T*) was genotyped in the BC1_LD animals and included in the eGWAS analysis. However, no significant association was found between this mutation and the *FADS2* mRNA expression. Further analyses are necessary to find new candidate *cis*-acting polymorphisms implicated in the regulation of *FADS2* gene expression.

Three regions on SSC6, SSC8, and SSC10 were associated in *trans* with *FADS2* gene expression where several lipid-related genes were identified (Table 2), highlighting the Lysine Demethylase 1A (*KDM1A*) gene, also known as *LSD1*, located on SSC6. This gene has been recently identified as a novel regulator of lipid metabolism as it is required for the expression of *SREBF1* and for the efficient binding of *SREBF1* to their target gene promoters^47^. Remarkably, *SREBF1* regulates the expression of desaturases^48^, suggesting the *KDM1A* gene as a potential candidate regulator of *FADS2* gene expression. Supporting this hypothesis, a transcription binding site for SREBF1 in the *FADS2* proximal promoter region was predicted by LASAGNA (Supplementary Table S4).

The most associated SNP for *FADS2 trans*-eQTL on SSC8 was an intronic polymorphism (MARC0041089; *p*-value = 1.93 × 10^−5^; â = 0.516) in the Insulin Like Growth Factor Binding Protein 7 (*IGFBP7*) gene (Supplementary Table S2). This gene encodes a member of the insulin-like growth factor (IGF)-binding protein and through their regulation of IGFs and insulin may influence the metabolism of adipocytes, having implications in the development of obesity and insulin resistance^49^.

#### The *SLC27A4* gene

The *SLC27A4* (Solute Carrier Family 27 (Fatty Acid Transporter), Member 4) gene expression GWAS revealed three *trans*-eQTL on SSC9, SSC14, and SSC15 (Supplementary Fig. S1B). On SSC9, the Prolylcarboxypeptidase (*PRCP*) gene was located. It is an important regulator of energy and glucose homeostasis^50^. For the SSC14 *trans*-eQTL, no strong candidate genes related to lipid metabolism could be detected. Finally, on SSC15, Phenylalanyl-tRNA Synthetase Beta Subunit (*FARSB*) and Monoacylglycerol O-Acyltransferase 1 (*MOGAT1*) lipid-related genes were also identified.

### Sex effect on gene expression in the adipose tissue

**Figure 2:**
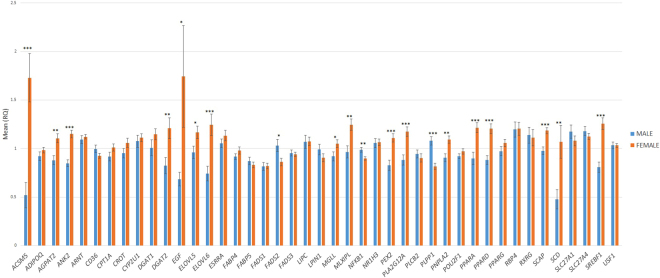
Comparison between males and females of gene expression levels of 43 lipid-related genes in adipose tissue. Data represent means ± standard error of the means (SEM). Significant differences between sexes are indicated as *p-value ≤ 0.05, **p-value ≤ 0.01 and ***p-value ≤ 0.001.

The control of FA homeostasis has been described to have a pronounced sexual dimorphism^51^. In agreement, sex differences in the control of gene expression have been identified in different tissues like muscle, liver, adipose tissue and brain in mice^52,53^. In the present work, a significant sex effect (*p*-value ≤ 0.05) on gene expression levels was detected in 20 out of the 43 genes analyzed (47%): *ACSM5*, *AGPAT2*, *ANK2*, *ATGL*, *DGAT2*, *EGF*, *ELOVL5*, *ELOVL6*, *FADS2*, *MGLL*, *MLXIPL*, *NFKB1*, *PEX2*, *PLA2G12A*, *PLPP1*, *PPARA*, *PPARD*, *SCAP*, *SCD*, and *SREBF1* (Fig. 2). Overall, a higher number of genes were more expressed in females. Conversely, only three genes (*FADS2*, *NFKB1*, and *PLPP1*) showed a higher expression in males (Supplementary Table S6). Among them, some key regulators of lipid metabolism such as *PPARA*, *SCD*, and *SREBF1* which may be determining the bias observed in the female over-expressed genes. In accordance, lipogenic genes such as *SCD* and *SREBF1* were also more expressed in females in the *Longissimus dorsi* muscle^15^ and liver^16^ of pigs. However, we observed a different pattern of expression for *PPARA*, showing a higher expression level in females in liver and adipose tissue and a higher expression in males in muscle^15,16^. Finally, for *ACSM5*, *AGPAT2*, *MLXIPL*, *EGF*, *ELOVL5*, *PEX2*, and *SCAP* the same female-biased gene expression as in pig liver tissue^16^ was observed. This agree with the results observed in mice, in which the analysis of genes differentially expressed between sexes in liver, adipose, muscle, and brain revealed common functionalities of steroid and lipid metabolism among the sexually dimorphic genes between liver and adipose tissues. Furthermore, the overlap of sexually dimorphic genes was higher between adipose and liver tissues (22.9%) than between adipose and muscle tissues (6.6%;^52^). The highest rate of coincidence of female-biased gene expression among liver and adipose tissue may suggest common regulatory mechanisms in both tissues associated with sex differences.

### Fatty acid composition and gene expression in the adipose tissue

To explore the relationship between gene expression levels and FA composition percentages in BF and muscle, Pearson’s correlation analyses were performed. In general moderate correlations were identified for BF (Supplementary Fig. S3) and muscle (Supplementary Fig. S4).

For the FA composition percentages in BF, the *CD36* gene, which may function as a regulator of FA transport^54^, showed a negative correlation with palmitoleic acid (C16:1(n-7)) (r = −0.31; *p*-value = 9.04 × 10^−4^). Palmitoleic acid (C16:1(n-7)) has been identified as a lipokine involved in the maintenance of systemic metabolic homeostasis^55^. In addition, a negative correlation between *PLPP1* gene expression and palmitic acid (C16:0) (r = −0.35; *p*-value = 1.46 × 10^−4^) was found. This gene is a member of the phosphatidic acid phosphatase (PAP) family and participates in the synthesis of glycerolipids. Moreover, *PLPP1* gene expression showed moderate correlations with the percentage of *cis-7* hexadecenoic (C16:1(n-9)) (r = 0.32; *p*-value = 5.40 × 10^−4^) and C20:3(n-9) (r = 0.33; *p*-value = 3.40 × 10^−4^) FAs.

The *cis-7* hexadecenoic acid (C16:1(n-9)) also showed negative correlation (r = −0.39; *p*-value = 2.05 × 10^−5^) with *SCD* gene expression, which plays an important role in the regulation of the expression of genes involved in lipogenesis^56^. In accordance, Fernández *et al*.^57^ have reported that *SCD* haplotypes, constituted by *SCDg.2108 C*>*T*, *g.2228* *T*>*C* and *G.2520* *G*>*A*, are associated with *SCD* gene expression in backfat and the fatty acid desaturation ratio in backfat and intramuscular fat in a Iberian x Duroc backcross.

Furthermore, the octadecenoic acid (C18:1(n-7)) was negatively correlated with *CROT* (r = −0.36; *p*-value = 1.02 × 10^−4^) and *USF1* (r = −0.35; *p*-value = 1.29 × 10^−4^) gene expressions. The *CROT* gene plays a role in the pathway of FA beta-oxidation, and provides a crucial step in the transport of medium and long-chain acyl-coA out of the mammalian peroxisome to the mitochondria^58^. Moreover, *USF1* gene is involved in the maintenance of high levels of FA synthase transcription under lipogenic conditions^59^.

For the FA composition percentages in muscle, *PLPP1* mRNA expression was moderately correlated with the percentage of α-linolenic acid (C18:3(n-3)) (r = 0.39; *p*-value = 2.18 × 10^−5^), the double bond index (DBI) (r = 0.31; *p*-value = 7.69 × 10^−4^), the unsaturation index (UI) (r = 0.35; *p*-value = 1.33 × 10^−4^), and the ratio PUFA/SFA (r = 0.31; *p*-value = 8.76 × 10^−4^). Negative correlations were also identified with palmitic acid (C16:0) (r = −0.39; *p*-value = 1.56 × 10^−5^). The great variability between the *PLPP1* gene expression and many FA composition percentages may be explained by its multiple functions, *PLPP1* converts phosphatidic acid to diacylglycerol, and function in *de novo* synthesis of glycerolipids as well as in receptor activated signal transduction mediated by phospholipase D^60^. Furthermore, this gene has been described as a catabolic enzyme for lysophosphatidic acid^61^, and has been identified in the enriched functional category from the lipid catabolic process as a highly expressed gene in a transcriptome analysis of Basque pigs^62^.

### Functional network analysis of genes mapping into eQTLs

To take a deep look at the regulatory mechanisms that are influencing the gene expression phenotypes, gene annotation of the 19 eQTLs chromosomal regions was performed. For *trans*-eQTLs all the genes located within ±1 Mb were selected for gene annotation. Conversely, for *cis*-eQTLs only the studied candidate gene was considered for further analyses. In the 19 eQTLs, a total of 474 protein-coding genes, 2 processed transcripts, 11 miRNAs, 3 miscRNAs, 10 pseudogenes, 6 rRNAs, 17 snoRNAs, and 21 snRNAs were annotated (Supplementary Table S5). From the 474 protein-coding genes with Ensembl Gene ID, 393 have at least one human orthologous gene (Supplementary Table S7) and were submitted to the Ingenuity Pathway Analysis software (IPA, Ingenuity Systems Inc.) to perform a functional categorization.

Several networks related to lipid metabolism were identified: (i) lipid metabolism, small molecule biochemistry, infectious diseases (score = 42; interval = 8); (ii) lipid metabolism, small molecule biochemistry, cellular assembly and organization (score = 32; interval = 8); (iii) carbohydrate metabolism, organ morphology, reproductive system development and function (score = 30; interval = 3), and (iv) amino acid metabolism, carbohydrate metabolism, molecular transport (score = 18; interval = 9) (Supplementary Table S8). Interestingly, two of the identified networks have the Akt complex as central (ID = 10 and 26; Supplementary Table S9). It is known that the PKB/Akt plays an important role in the insulin regulation of glucose transport^63^. In addition, it has been also identified as central in the main over-represented pathways in a muscle transcriptome study between individuals phenotypically extreme for IMFA composition^64^, in a muscle eQTL analysis of 45 lipid-related genes^15^, and in a liver eQTL analysis of 44 candidate genes related with lipid metabolism^16^. Remarkably, several of the potential regulators annotated in *trans*-eQTL for *FABP4* and *FADS2* (*BSCL2*, *FADS1*, *FADS2*, *KDM1A*, *LGALS12*, *PLA2G16*, *PLB1*, and *PPP1CB*) were identified inside the Akt pathway. These findings suggest this pathway as central in the genetic determination of FA composition traits in pigs.

### Prediction analysis of transcription factor binding sites

To identify master regulators of global gene expression in all the genes analyzed in the present study, an *in silico* identification of the TF binding sites in the promoter region of (1) the 415 genes with human orthologous ID annotated in the *trans*-eQTL intervals and, (2) the 43 candidate lipid-related genes was performed using the iRegulon Cytoscape plugin^65^. The *PPARG* gene was the most enriched TF motif (normalized enrichment score, NES = 5.134; 167 target genes, 30 out of the 44 analyzed lipid-related candidate genes; Supplementary Table S9). This is in accordance with the fact that this gene is an adipogenic TF considered an important regulator of lipid and carbohydrate metabolism^66^. In our analysis the mRNA expression of *PPARG* was highly correlated with the mRNA expression of genes implicated in lipid transport, lipogenesis and TG synthesis (Supplementary Table S10), suggesting an important role of this TF in lipid storage. Interestingly, this TF has been identified as a key regulator of FA composition in the same animal material using a co-association network analysis^19^ and in a muscle eQTL analysis of 45 lipid-related genes^15^.

## Conclusions

In the present study, potential candidate genes and genetic variants regulating the expression of lipid-related genes associated with meat quality traits in pigs have been identified by eGWAS analysis in the backfat adipose tissue of Iberian x Landrace backcrossed pigs. While some genetic variants identified in *cis* seem to be tissue specific (*FADS2*, *FABP4*), other may exert a similar regulatory control in different tissues (*ACSM5*). In addition to the well-known transcription factors SREBF1 and PPARs, that regulate the expression of lipogenic genes, a *trans*-eQTL on SSC13 identified for the *ELOVL6* gene was associated with the expression of *ELOVL5* and *SCD* genes, which are implicated in the elongation and desaturation of fatty acids, supporting the involvement of common elements regulating their mRNA expression in adipose tissue. These results increase our knowledge of the regulatory mechanisms implicated in adipose tissue lipid metabolism and its consequences in lipid-related traits.

## Methods

### Ethics Statement

All animal procedures were performed according to the Spanish Policy for Animal Protection RD1201/05, which meets the European Union Directive 86/609 about the protection of animals used in experimentation. Animals were sacrificed in a commercial slaughterhouse following national and institutional guidelines for the Good Experimental Practices and approved by the Ethical Committee of the Institution (IRTA- Institut de Recerca i Tecnologia Agroalimentàries).

### Animal Samples

The IBMAP resource population was used in this study. This population was established by crossing 3 Iberian (Guadyerbas line) boars with 31 Landrace sows^67^, and 5 F_1_ males and 25 Landrace sows were crossed to obtain the BC1_LD generation (25% Iberian × 75% Landrace). The Iberian pig breed is characterized by high levels of IMF with higher MUFA and SFA content and an extremely fat body composition. In contrast, the Landrace breed has an efficient meat production, with low IMF and high PUFA content, and with a rapid growth and leaner carcass. Hence, the IBMAP experimental cross was generated to study the genetic basis of growth and meat quality traits differentiating the two breeds^68^. Here, we report results based on 115 BC1_LD pigs. All animals were maintained under intensive conditions and feeding was *ad libitum* with a cereal-based commercial diet. Backcross animals were slaughtered at an average age of 179.9 ± 8.9 days, and samples of BF tissue were collected, snap-frozen in liquid nitrogen and stored at −80 °C until analysis. Genomic DNA was extracted from diaphragm samples of all animals by the phenol-chloroform method^69^.

### SNP Genotyping and quality control

A total of 115 animals belonging to BC1_LD were genotyped for 62,163 SNPs with the *PorcineSNP60 Beadchip*^70^ following the Infinium HD Assay Ultra protocol (*Illumina* Inc.; San Diego, USA). Raw data was visualized with GenomeStudio software (*Illumina* Inc.; San Diego, USA) and trimmed for high genotyping quality (call rate > 0.99). Plink software^71^ was used to remove markers that showed a minor allele frequency (MAF) less than 5% and SNPs with more than 5% of missing genotypes. The SNPs not mapped in the *Sscrofa10.2* assembly were also excluded. After the quality control filter, a subset of 40,460 SNPs remained.

In addition, ten polymorphisms were genotyped in the BC1_LD animals: two SNPs located in the proximal promoter region of the *ACSM5* (*g.26260422* *G*>*A*, rs331702081^15^) and the *FADS2* (rs331050552) genes, and one indel and one SNP located in the intron 1 (*FABP4:g.2634_2635insC*^72^) and in the 3′UTR region of the *FABP4* gene (*FABP4:g.6723* *A*>*G*), respectively. For the *ELOVL6* gene, three SNPs located in the promoter region (*ELOVL6:c.-533C*>*T*, *ELOVL6:c.-480C*>*T*, and *ELOVL6:c.-394G*>*A*^18,29^), one in exon 4 (*ELOVL6:c.416* *C*>*T*^18^) and two in the 3′UTR region (*ELOVL6:c.1408* *A*>*G* and *ELOVL6:c.1922C*>*T*^29^) were genotyped. *ACSM5* and *FADS2* SNPs were genotyped using Taqman OpenArray^™^ genotyping plates designed in a QuantStudio^™^ 12 K flex Real-Time PCR System (*ThermoFisher Scientific*) using the SVGM platform (http://svgm.es/ca/Platforms/). The pyrosequencing protocol described by Mercadé *et al*.^72^ and the High Resolution Melting methodology (HRM, *ThermoFisher Scientific*) were used for genotyping the indel and the SNP of *FABP4* gene, respectively. The SNPs belonging to *ELOVL6* gene were genotyped using the KASP SNP genotyping service from LGC (http://www.lgcgroup.com/genotyping/).

### Characterization of the 3′UTR of porcine *FABP4* gene

The 3′UTR of the *FABP4* gene was amplified and sequenced in 10 animals with extreme values of *FABP4* mRNA expression in BF.

The 3′UTR variants of *FABP4* gene were characterized by 3′-RACE PCR using UAP reverse primer and, FABP4-3NC-1-Fw and FABP4-3NC-2-Fw forward primers (Supplementary Table S11). The specific bands were excised from agarose gel and purified using NucleoSpin^®^ Gel and PCR Clean-up (Macherey-Nagel GmbH & Co. KG) and sequenced in forward and reverse directions.

All primers were designed using PRIMER3 software^73^ based on the Y16039 sequence^33^ and validated using PRIMER EXPRESS^™^ (Applied Biosystems). In all cases, PCR was performed in a 25 µl volume containing 2 µl of cDNA. PCR reaction contained 0.6 units of AmpliTaq Gold (Applied Biosystems), 2.5 mM MgCl_2_, 0.2 mM of each dNTP and 0.5 µM of each primer. PCR were carried out under the following conditions: 94 °C for 10 min, 35 cycles of 94 °C for 1 min, 62 °C for 1 min and 72 °C for 1 min, with a final extension at 72 °C for 7 min. Purification was performed using the ExoSAP-IT^®^ method and sequenced with a Big Dye Terminator v.3.1 Cycle Sequencing Kit in an ABI 3730 analyzer (Applied Biosystems). Polymorphisms were identified using SeqScape v2.5 program (Applied Biosystems).

miRDB^74^ program was run to find putative target miRNAs in the 3′UTR region of *FABP4*. For this purpose, the human miRNA database was used.

### Detection of polymorphisms in the promoter region of the *FADS2* gene

Polymorphisms in the proximal promoter region of the *FADS2* gene were identified from the whole-genome sequence data of seven founders of the IBMAP experimental population (SRA accession numbers: SRR5229970, SRR5229971, SRR5229972, SRR5229973, SRR5229974, SRR5229975, and SRR5229976) using the *Integrative Genomics Viewer* (*IGV*) *software*^46^.

### Gene expression profiling

Total RNA was isolated from the BF of the 115 BC1_LD samples with RiboPure^™^ RNA Purification Kit (Ambion; Austin, TX, USA). Total RNA was quantified in a NanoDrop ND-1000 spectrophotometer (NanoDrop products; Wilmington, DE, USA). The RNA was converted to cDNA using the *High-Capacity cDNA Reverse Transcription kit* (Applied Biosystems) in 20 µl of reactions, following the manufacturer’s instructions. The cDNA samples were loaded into a *Dynamic Array* 48.48 chip in a BioMark system (Fluidigm; San Francisco, CA, USA) through an integrated fluidic circuit controller following a protocol previously described^19^.

For this experiment, the expressed levels of 48 genes were analyzed: 44 target genes and four reference genes (*ACTB*, *B2M*, *HPRT1*, and *TBP*). The *ACTB* and *TBP* were the two most stable reference genes and were used to normalize the expression levels of the target genes. Primers used for the analysis were designed using PrimerExpress 2.0 software (Applied Biosystems) and are detailed in Supplementary Table S12. Data was collected using the Fluidigm Real-Time PCR analysis software 3.0.2 (Fluidigm) and analyzed using the DAG expression software 1.0.5.5^75^ applying the relative standard curve method. Samples targeted in this study were analyzed in duplicate. The normalized quantity (NQ) values of each sample and assay were used to compare our data. Data obtained were normalized by performing log_2_ transformation of the NQ value. The sex effect was also tested by using a linear model with the *lm* function of R program^76^.

### Gene expression association analysis

In order to detect SNPs associated with gene expression levels, eGWAS was performed using as phenotype the expression values of 43 genes in adipose tissue. A mixed model (1) was employed in Qxpak 5.0^77^:

1 $${{\rm{Y}}}_{{\rm{ijk}}}{=\mathrm{Sex}}_{{\rm{i}}}{+\mathrm{Batch}}_{{\rm{j}}}{+{\rm{\lambda }}}_{{\rm{l}}}{{\rm{a}}}_{{\rm{l}}}{+{\rm{u}}}_{{\rm{k}}}{+{\rm{e}}}_{{\rm{ijkl}}},$$

in which Y_ijk_ was the k^th^ individual expression record of each one of the 43 analyzed genes, sex (two levels) and batch (corresponding to the slaughterhouse group; five levels) were fixed effects, λ_l_ was a −1 (aa), 0 (Aa), +1 (AA) indicator variable depending on the k^th^ individual’s genotype for the l^th^ SNP, a_l_ represents the additive effect associated with the l^th^ SNP, u_k_ is the infinitesimal genetic effect with random distribution N(**0**, **A**σ_u_^2^) where **A** is the numerator of the pedigree-based relationship matrix and the e_ijkl_ the residual.

The association analyses of the *ACSM5* (rs331702081), *ELOVL6* (*ELOVL6:c.-533C>T*, *ELOVL6:c.-480C>T*, *ELOVL6:c.-394G>A*, *ELOVL6:c.416 C>T*, *ELOVL6:c.1408 A>G*, and *ELOVL6:c.1922C>T*), *FABP4* (*FABP4:g.2634_2635insC* and *FABP4:g.6723 A>G*), and *FADS2* (rs331050552) polymorphisms with the *ACSM5*, *ELOVL6*, *FABP4*, and *FADS2* mRNA expression, respectively, were performed using the same mixed model described above.

The R package *q*-value was used to calculate the false discovery rate (FDR), and the cut-off of the significant association at the whole genome level was set at the *q*-value ≤ 0.05^77,78^, meaning that 5% of all significant *p*-values will be false positives.

The identified SNPs were classified as *cis* when they were at 5 Mb upstream or downstream of the gene position and as *trans* when they were located elsewhere in the genome. Significant associated SNPs located less than 10 Mb apart were considered as belonging to the same interval.

### Gene annotation and functional classification

The significantly associated SNPs (FDR ≤ 0.05) identified were mapped in the *Sscrofa10.2* assembly and were annotated with the Ensembl Genes 84 Database using VEP software^79^.

The genomic eQTL intervals were annotated using Biomart software [http://www.biomart.org] adding a genomic region of one Mb from the start and end of each *trans*-eQTLs. Only the studied candidate gene was annotated for *cis*-eQTLs.

The *Core Analysis* function included in IPA was used to perform the functional analysis of genes mapped in the 19 eQTL regions. This software was used for data interpretation in the context of biological processes, pathways and networks. All information generated is derived from the Ingenuity Pathway Knowledge Base (IPKB), which is based on functions and interactions of genes published in the literature. RNA-Seq data of BF from BC1_LD individuals^17^ and Gene Expression Atlas^80^ were used to determine which of the lipid-related genes annotated in the genomic eQTL intervals were expressed in adipose tissue. Finally, a prediction analysis of TF binding sites was performed in the promoter region of the annotated genes. The iRegulon Cytoscape plugin^65^ was used to analyze the TFs and their related target genes. iRegulon relies on the analysis of the regulatory sequences around each gene, and use databases of nearly 10,000 TF motifs and 1,000 ChIP-seq data sets or “tracks”. In the present study, a high NES threshold value (NES > 5) was considered for the selection of potential relationships (the default NES cut-off in iRegulon is 3). A high NES score indicates (1) a motif that recovers a large proportion of the input genes within the top of its ranking and (2) higher reliability.

### Gene expression and correlation analysis with phenotypes

A correlation analysis was performed to explore the association among the expressions of the 43 genes. Moreover, to analyze the relationships between gene expression and phenotypes, Pearson’s correlations among gene expression and FA composition percentages in BF^6^ and *Longissimus dorsi* muscle^5^ were performed. If required, data was normalized applying the log_2_ transformation. Then, gene expression and the composition of FA in BF and muscle were corrected both by gender (two levels) and batch effects (five levels), and the composition of FA traits was also adjusted for carcass weight, using the glm R package^81^. The remaining residuals of the phenotypes and gene expression values corrected for the corresponding effects were used to obtain the pairwise correlations. The hierarchical clustering option of PermutMatrix software^82^ was used to visualize the results of both traits and genes.

## Electronic supplementary material

Supplementary Information 

Supplementary Table S5
