# Supplementary material for: Expression analysis of candidate genes for fatty acid composition in adipose tissue and identification of regulatory regions
Source: Sci Rep. 2018 Feb 1;8:2045. doi: 10.1038/s41598-018-20473-3 (PMC5794915; doi:10.1038/s41598-018-20473-3)
Supplement: Supplementary file 1 — Supplementary Information [file 41598_2018_20473_MOESM1_ESM.pdf]

**SUPPLEMENTARY MATERIAL**

# Expression analysis of candidate genes for fatty acid composition in adipose tissue and identification of regulatory regions

Manuel Revilla[1,2]*, Anna Puig-Oliveras[1,2], Daniel Crespo-Piazuelo[1,2], Lourdes Criado-Mesas[1,2], Anna Castelló[1,2], Ana I Fernández[3], Maria Ballester[4†], Josep M Folch[1,2†]

[1] Animal Genomics Department, Centre for Research in Agricultural Genomics (CRAG), CSIC-IRTA-UAB-UB, Campus UAB, 08193 Bellaterra, Spain

[2] Departament de Ciència Animal i dels Aliments, Facultat de Veterinària, Universitat Autònoma de Barcelona (UAB), 08193 Bellaterra, Spain

[3] Departamento de Genética Animal, Instituto Nacional de Investigación y Tecnología Agraria y Alimentaria (INIA), 28040 Madrid, Spain

[4] Departament de Genètica i Millora Animal, Institut de Recerca i Tecnologia Agroalimentàries (IRTA), Torre Marimon, 08140 Caldes de Montbui, Spain

* E-mail: manuel.revilla@cragenomica.es

† Equal contribution

**Figure S1.** GWAS plot of *ELOVL6* and *SLC27A4A* gene expression in adipose tissue. The X-axis represents chromosome positions in Mb relative to *Sscrofa10.2* assembly of the pig genome and the Y-axis shows the –log10 (*p*-value). Horizontal dashed lines indicate the genome-wide significance level. Plot of eGWAS for (**A**) *ELOVL6*, and (**B**) *SLC27A4* gene expression in backfat.

**Figure S2.** Associations of SNPs from SSC8 and *ELOVL6* polymorphisms for *ELOVL6* gene expression in backfat. The X-axis represents positions of SSC8 in Mb relative to *Sscrofa10.2* assembly of the pig genome and the Y-axis shows the –log10 (*p*-value). Horizontal dashed lines indicate the chromosome significance level.

**Figure S3.** Clustered heat map to visualize correlations among gene expression levels (NQ) of the 43 genes and fatty content in adipose tissue. Color legend was adjusted to minimal and maximal values to differentiate the differences.

**Figure S4.** Clustered heat map to visualize correlations among gene expression levels (NQ) of the 43 genes and fatty content in muscle. Color legend was adjusted to minimal and maximal values to differentiate the differences.

**Table S1.** List of 44 lipid-related genes selected to study their expression in the present study. The overlapping between categories is labelled by colors.

**Table S2.** Description of the 193 significant expression-associated SNPs (eSNPs).

**Table S3.** Transcription factor binding sites for *PPARG* in the *ACSM5* promoter region gene.

**Table S4.** Transcription factor binding site for *SREBF1* in the *FADS2* promoter region gene.

**Table S5.** Gene annotation of genes within the eQTL intervals. Annotation was performed by considering for *trans*-eQTLs the eQTL interval ±1 Mb; whereas for *cis*-eQTLs only the studied gene was selected (*ACSM5, FABP4*, and *FADS2*).

**Table S6.** Mean comparison between males and females of backfat gene expression levels of 43 lipid-related genes.

**Table S7.** Description of the 393 human orthologous genes.

**Table S8.** Top functional networks and molecules identified with IPA form the list of annotated genes mapping within the 19 eQTLs.

**Table S9.** Transcription factor binding sites for the *PPARG* gene.

**Table S10.** Pearson's correlations of *PPARG* mRNA expression and analyze-related genes.

**Table S11.** Primers used for the characterization of the 3'UTR of porcine *FABP4* gene.

**Table S12.** Primers used for the analyses of gene expression of the 48 genes by real-time PCR.

**Figure S1.** GWAS plot of *ELOVL6* and *SLC27A4A* gene expression in adipose tissue. The X-axis represents chromosome positions in Mb relative to *Sscrofa10.2* assembly of the pig genome and the Y-axis shows the –log10 (*p*-value). Horizontal dashed lines indicate the genome-wide significance level. Plot of eGWAS for (**A**) *ELOVL6*, and (**B**) *SLC27A4* gene expression in backfat.

**A)**

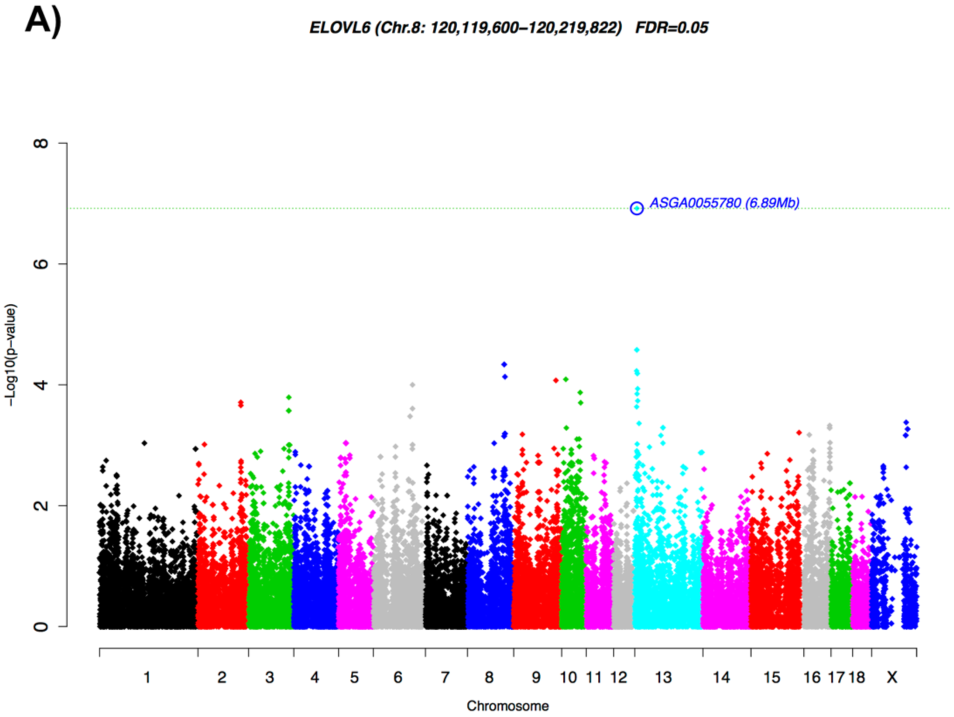


**B)**

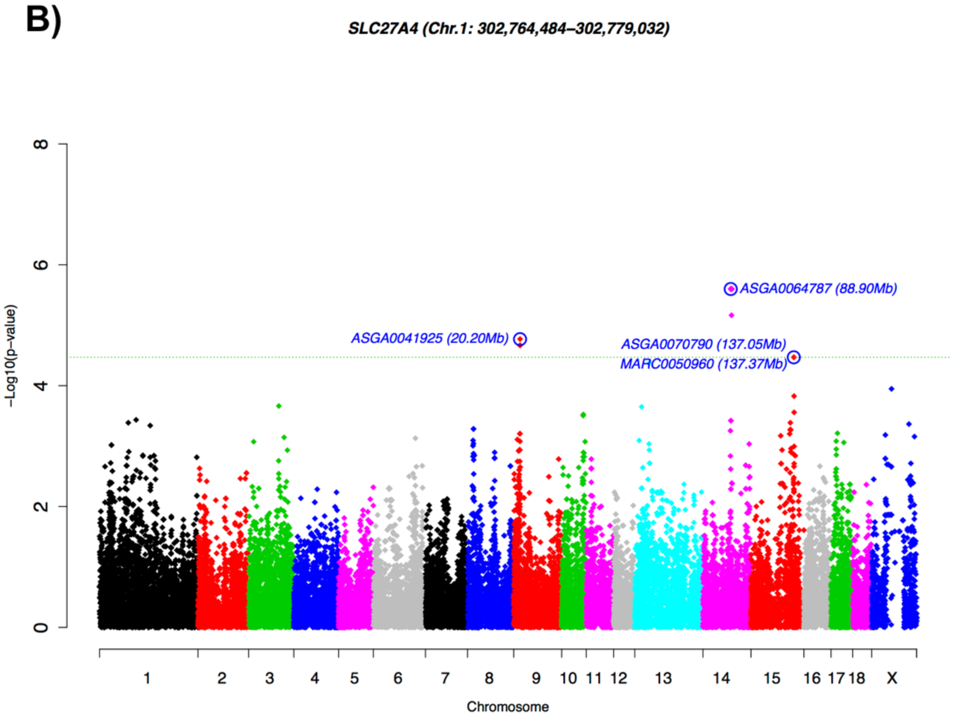

**Figure S2.** Associations of SNPs from SSC8 and *ELOVL6* polymorphisms for *ELOVL6* gene expression in backfat. The X-axis represents positions of SSC8 in Mb relative to *Sscrofa10.2* assembly of the pig genome and the Y-axis shows the –log10 (*p*-value). Horizontal dashed lines indicate the chromosome significance level.

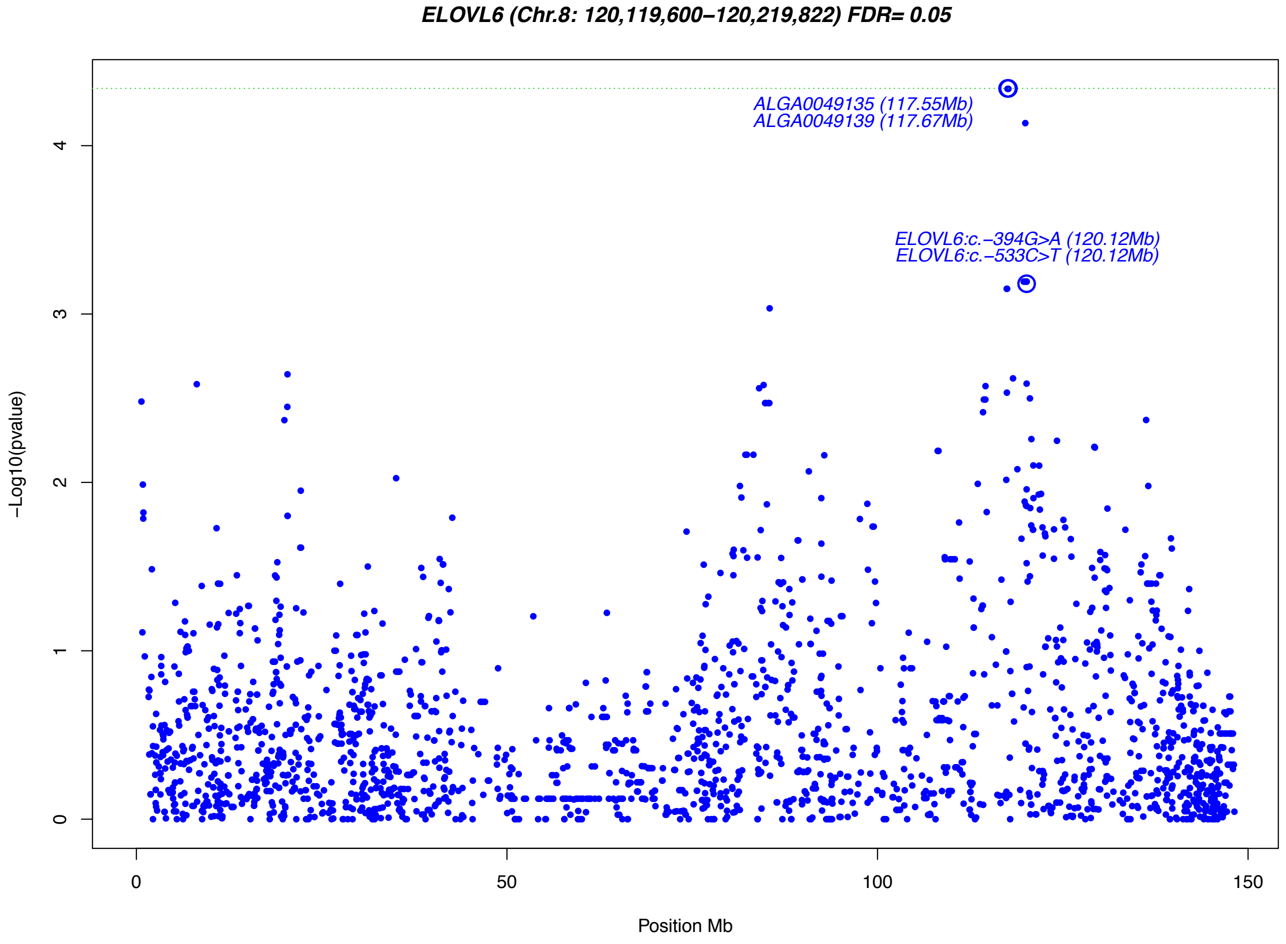

**Figure S3.** Clustered heat map to visualize correlations among gene expression levels (NQ) of the 43 genes and fatty content in adipose tissue. Color legend was adjusted to minimal and maximal values to differentiate the differences.

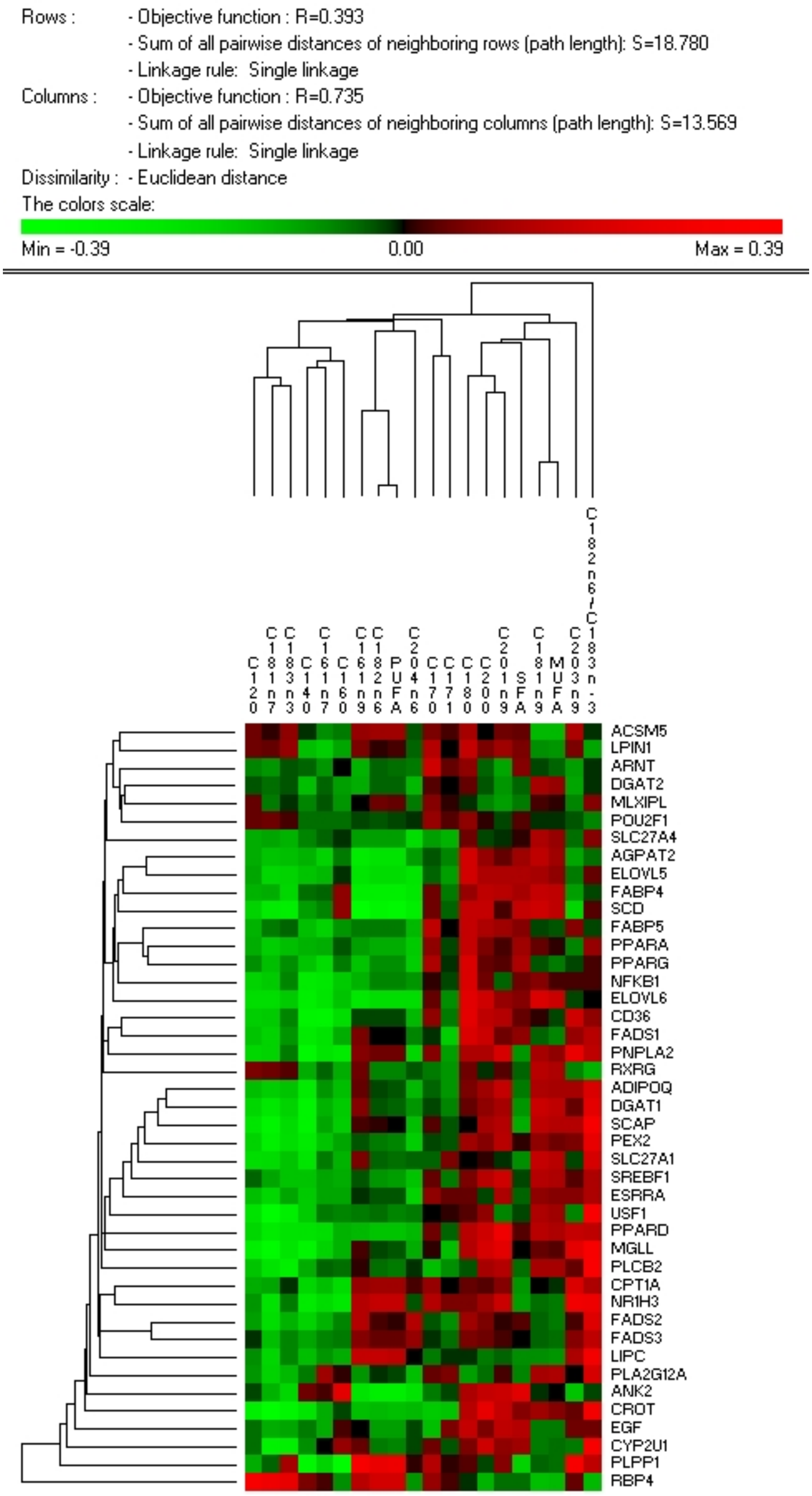

**Figure S4.** Clustered heat map to visualize correlations among gene expression levels (NQ) of the 43 genes and fatty content in muscle. Color legend was adjusted to minimal and maximal values to differentiate the differences.

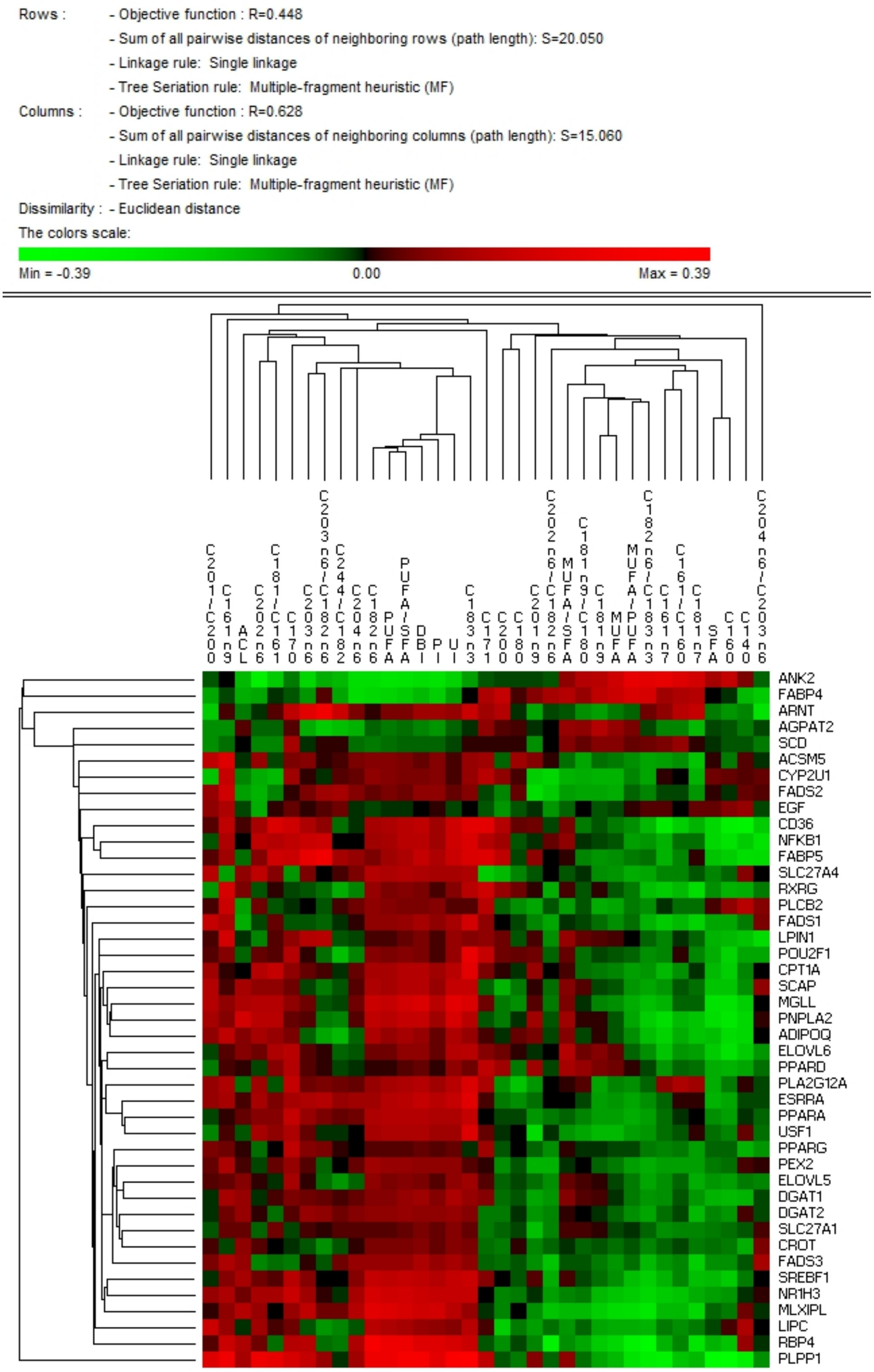

**Table S1.** List of 44 lipid-related genes selected to study their expression in the present study. The overlapping between categories is labelled by colors.

| Description | Bibliographic reference | Genes |
|---|---|---|
| Candidate functional and positional genes identified in a GWAS study for intramuscular FA composition in the BC1_LD backcross | [5] | *ARNT*, *CYP2U1*, *EGF*, *ELOVL6*, *FABP4*, *FABP5*, *FADS1*, *FADS2*, *FADS3*, *NFKB1*, *PLA2G12A*, *PLCB2*, *PLPP1* and *USF1* |
| Genes differentially expressed by RNA-Seq in the adipose tissue of two phenotypically extreme groups of animals for intramuscular FA composition in the BC1_LD backcross | [17] | *ELOVL6* and *SCD* |
| Candidate positional gene affecting palmitic and palmitoleic FA in pigs | [18] | *ELOVL6* |
| Genes related to lipid metabolism identified in gene co-association networks for FA composition in the BC1_LD backcross | [19] | *ACSM5*, *ANK2*, *ARNT*, *FABP4*, *FABP5*, *MGLL* and *PPARG* |
| Genes related to lipid metabolism identified in gene co-association networks for fatness and growth related traits in the IBMAP cross | [20] | *ARNT* and *PPARG* |
| Candidate positional genes identified in a GWAS study for backfat FA composition in the BC1_LD backcross | [21] | *FADS1*, *FADS2* and *FADS3* |
| Transporters involved in lipid metabolism | | *RBP4*, *SCAP*, *SLC27A1* and *SLC27A4* |
| Enzymes involved in lipid metabolism | | *AGPAT2*, *CPT1A*, *CROT*, *DGAT1*, *DGAT2*, *ELOVL5*, *LIPC*, *LPIN1*, *PEX2* and *PNPLA2* |
| Transcriptional factors, cofactors or nuclear receptors involved in lipid metabolism | | *CD36*, *ESRRA*, *MLXIPL*, *NR1H3*, *POU2F1*, *PPARA*, *PPARD*, *PPARGC1A*, *RXRG* and *SREBF1* |
| Adipokine of white fat tissue | [22] | *ADIPOQ* |

**Table S2.** Description of the 193 significant expression-associated SNPs (eSNPs).

| Interval | Chr | SNP | Position (bp) | fA1 | fA2 | *p*-value | *q*-value | Associated Gene | Consequence | Ensembl_GeneId | Gene Id | *Cis/Trans*-eSNPs |
|---|---|---|---|---|---|---|---|---|---|---|---|---|
| I1 | 2 | ASGA0093797 | 134992155 | 0.94 | 0.06 | $1.55x10^{-6}$ | $5.48x10^{-4}$ | *ACSM5* | intergenic | - | - | *trans* |
| I2 | 3 | ASGA0089930 | 16473829 | 0.74 | 0.26 | $4.45x10^{-6}$ | $1.30x10^{-3}$ | *ACSM5* | intergenic | - | - | *trans* |
| I2 | 3 | H3GA0053939 | 18724340 | 0.90 | 0.10 | $3.75x10^{-6}$ | $1.17x10^{-3}$ | *ACSM5* | intronic | ENSSSCG00000007804 | *SH2B1* | *trans* |
| I2 | 3 | ASGA0101457 | 18742778 | 0.90 | 0.10 | $3.03x10^{-7}$ | $1.22 x10^{-4}$ | *ACSM5* | intronic | ENSSSCG00000007805 | *ATP2A1* | *trans* |
| I2 | 3 | ALGA0112844 | 19373384 | 0.67 | 0.33 | $3.66x10^{-8}$ | $1.84x10^{-5}$ | *ACSM5* | intronic | ENSSSCG00000022785 | *GSG1L* | *trans* |
| I2 | 3 | ASGA0098276 | 21286903 | 0.82 | 0.18 | $1.69x10^{-14}$ | $1.70x10^{-11}$ | *ACSM5* | intergenic | - | - | *cis* |
| I2 | 3 | ALGA0017974 | 21309417 | 0.77 | 0.23 | $1.22x10^{-15}$ | $1.51x10^{-12}$ | *ACSM5* | intergenic | - | - | *cis* |
| I2 | 3 | ASGA0013836 | 21387300 | 0.77 | 0.23 | $6.97x10^{-10}$ | $4.76x10^{-7}$ | *ACSM5* | intergenic | - | - | *cis* |
| I2 | 3 | ALGA0017987 | 21528275 | 0.53 | 0.47 | $2.68x10^{-5}$ | $6.75x10^{-3}$ | *ACSM5* | intergenic | - | - | *cis* |
| I2 | 3 | ASGA0013852 | 21562254 | 0.73 | 0.27 | $5.69x10^{-8}$ | $2.79x10^{-5}$ | *ACSM5* | intergenic | - | - | *cis* |
| I2 | 3 | ALGA0123606 | 21777886 | 0.63 | 0.37 | $7.13x10^{-11}$ | $5.66x10^{-8}$ | *ACSM5* | intergenic | - | - | *cis* |
| I2 | 3 | ASGA0089383 | 22771385 | 0.50 | 0.50 | $1.19 x10^{-10}$ | $8.93x10^{-8}$ | *ACSM5* | intronic | ENSSSCG00000007831 | *CACNG3* | *cis* |
| I2 | 3 | MARC0101247 | 22772065 | 0.88 | 0.12 | $1.96x10^{-6}$ | $6.52x10^{-4}$ | *ACSM5* | intronic | ENSSSCG00000007831 | *CACNG3* | *cis* |
| I2 | 3 | ASGA0094123 | 22794781 | 0.84 | 0.16 | $7.97x10^{-6}$ | $2.23x10^{-3}$ | *ACSM5* | intronic | ENSSSCG00000007831 | *CACNG3* | *cis* |
| I2 | 3 | ALGA0018006 | 23015607 | 0.70 | 0.30 | $1.08x10^{-7}$ | $4.86x10^{-5}$ | *ACSM5* | intergenic | - | - | *cis* |
| I2 | 3 | ALGA0018040 | 23224453 | 0.66 | 0.34 | $3.33x10^{-16}$ | $4.39x10^{-13}$ | *ACSM5* | intergenic | - | - | *cis* |
| I2 | 3 | ASGA0098738 | 23442184 | 0.73 | 0.27 | $1.11x10^{-17}$ | $1.57x10^{-14}$ | *ACSM5* | downstream | ENSSSCG00000007836 | *SCNN1G* | *cis* |
| I2 | 3 | ALGA0103397 | 23522898 | 0.73 | 0.27 | $1.11x10^{-17}$ | $1.57x10^{-14}$ | *ACSM5* | upstream | ENSSSCG00000030424 | - | *cis* |
| I2 | 3 | ALGA0123533 | 23592793 | 0.60 | 0.40 | $7.20x10^{-11}$ | $5.66x10^{-8}$ | *ACSM5* | intergenic | - | - | *cis* |
| I2 | 3 | MARC0041570 | 23603118 | 0.73 | 0.27 | $1.11x10^{-17}$ | $1.57x10^{-14}$ | *ACSM5* | downstream | ENSSSCG00000026544 | *USP31* | *cis* |
| I2 | 3 | ASGA0093175 | 23778733 | 0.80 | 0.20 | $1.11x10^{-17}$ | $1.57x10^{-14}$ | *ACSM5* | intronic | ENSSSCG00000007837 | *HS3ST2* | *cis* |
| I2 | 3 | MARC0003844 | 23802761 | 0.80 | 0.20 | $1.11x10^{-17}$ | $1.57x10^{-14}$ | *ACSM5* | intronic | ENSSSCG00000007837 | *HS3ST2* | *cis* |
| I2 | 3 | MARC0047020 | 23961903 | 0.73 | 0.27 | $1.11x10^{-17}$ | $1.57x10^{-14}$ | *ACSM5* | intergenic | - | - | *cis* |

| Interval | Chr | SNP | Position (bp) | fA1 | fA2 | *p*-value | *q*-value | Associated Gene | Consequence | Ensembl_GeneId | Gene Id | *Cis/Trans*-eSNPs |
|---|---|---|---|---|---|---|---|---|---|---|---|---|
| I2 | 3 | ASGA0103399 | 24010664 | 0.75 | 0.25 | $1.11x10^{-17}$ | $1.57x10^{-14}$ | *ACSM5* | downstream | ENSSSCG00000007838 | *OTOA* | *cis* |
| I2 | 3 | ALGA0112358 | 24025177 | 0.80 | 0.20 | $4.93x10^{-11}$ | $4.10x10^{-8}$ | *ACSM5* | intronic | ENSSSCG00000007838 | *OTOA* | *cis* |
| I2 | 3 | ALGA0123020 | 24042892 | 0.78 | 0.22 | $7.14x10^{-13}$ | $6.50x10^{-10}$ | *ACSM5* | intronic | ENSSSCG00000007838 | *OTOA* | *cis* |
| I2 | 3 | ASGA0094818 | 24086731 | 0.33 | 0.67 | $2.69x10^{-05}$ | $6.75x10^{-3}$ | *ACSM5* | intergenic | - | - | *cis* |
| I2 | 3 | ALGA0115152 | 24218664 | 0.53 | 0.47 | $2.08x10^{-07}$ | $8.84x10^{-5}$ | *ACSM5* | intergenic | - | - | *cis* |
| I2 | 3 | ASGA0094620 | 24288043 | 0.71 | 0.29 | $2.22x10^{-15}$ | $2.43x10^{-12}$ | *ACSM5* | 3_prime_UTR_variant | ENSSSCG00000007839 | *EEF2K* | *cis* |
| I2 | 3 | ASGA0089883 | 24297157 | 0.71 | 0.29 | $2.22x10^{-15}$ | $2.43x10^{-12}$ | *ACSM5* | intronic | ENSSSCG00000007839 | *EEF2K* | *cis* |
| I2 | 3 | ASGA0099261 | 24299990 | 0.84 | 0.16 | $6.93x10^{-14}$ | $6.47x10^{-11}$ | *ACSM5* | intronic | ENSSSCG00000007839 | *EEF2K* | *cis* |
| I2 | 3 | ALGA0103433 | 24365103 | 0.40 | 0.60 | $4.04x10^{-05}$ | $9.20x10^{-3}$ | *ACSM5* | intronic | ENSSSCG00000007839 | *EEF2K* | *cis* |
| I2 | 3 | MARC0006151 | 24387052 | 0.47 | 0.53 | $2.70x10^{-05}$ | $6.75x10^{-3}$ | *ACSM5* | intronic | ENSSSCG00000007839 | *EEF2K* | *cis* |
| I2 | 3 | ASGA0101242 | 24418411 | 0.77 | 0.23 | $1.11x10^{-17}$ | $1.57x10^{-14}$ | *ACSM5* | downstream | ENSSSCG00000007842 | *SDR42E2* | *cis* |
| I2 | 3 | ASGA0013894 | 24602938 | 0.53 | 0.47 | $4.46x10^{-08}$ | $2.22x10^{-5}$ | *ACSM5* | upstream | ENSSSCG00000027510 | - | *cis* |
| I2 | 3 | MARC0110831 | 25162049 | 0.84 | 0.16 | $6.93x10^{-14}$ | $6.47x10^{-11}$ | *ACSM5* | intronic | ENSSSCG00000007847 | - | *cis* |
| I2 | 3 | MARC0115165 | 25531453 | 0.80 | 0.20 | $4.93x10^{-11}$ | $4.10x10^{-8}$ | *ACSM5* | upstream | ENSSSCG00000007849 | *CRYM* | *cis* |
| I2 | 3 | ASGA0085542 | 25695049 | 0.73 | 0.27 | $1.11x10^{-17}$ | $1.57x10^{-14}$ | *ACSM5* | intergenic | - | - | *cis* |
| I2 | 3 | MARC0060700 | 25794656 | 0.67 | 0.33 | $1.11x10^{-16}$ | $1.57x10^{-13}$ | *ACSM5* | intergenic | - | - | *cis* |
| I2 | 3 | ASGA0013904 | 25862724 | 0.74 | 0.26 | $1.11x10^{-17}$ | $1.57x10^{-14}$ | *ACSM5* | intergenic | - | - | *cis* |
| I2 | 3 | ASGA0013906 | 25939787 | 0.73 | 0.27 | $1.11x10^{-17}$ | $1.57x10^{-14}$ | *ACSM5* | upstream | ENSSSCG00000007853 | *LYRM1* | *cis* |
| I2 | 3 | ALGA0018079 | 25957412 | 0.73 | 0.27 | $1.11x10^{-17}$ | $1.57x10^{-14}$ | *ACSM5* | intergenic | - | - | *cis* |
| I2 | 3 | ASGA0095840 | 25990903 | 0.74 | 0.26 | $1.11x10^{-17}$ | $1.57x10^{-14}$ | *ACSM5* | intronic | ENSSSCG00000007855 | - | *cis* |
| I2 | 3 | MARC0108510 | 26002460 | 0.74 | 0.26 | $1.11x10^{-17}$ | $1.57x10^{-14}$ | *ACSM5* | intronic | ENSSSCG00000007855 | - | *cis* |
| I2 | 3 | ALGA0108097 | 26045382 | 0.74 | 0.26 | $1.11x10^{-17}$ | $1.57x10^{-14}$ | *ACSM5* | intronic | ENSSSCG00000007857 | *ACSM3* | *cis* |
| I2 | 3 | ASGA0103234 | 26183486 | 0.66 | 0.34 | $8.18x10^{-12}$ | $7.28x10^{-9}$ | *ACSM5* | intronic | ENSSSCG00000026453 | *ACSM5* | *cis* |
| I2 | 3 | ASGA0085560 | 26316304 | 0.76 | 0.24 | $1.11x10^{-17}$ | $1.57x10^{-14}$ | *ACSM5* | intergenic | - | - | *cis* |
| I2 | 3 | H3GA0053928 | 26392557 | 0.71 | 0.29 | $3.40x10^{-14}$ | $3.34x10^{-11}$ | *ACSM5* | intergenic | - | - | *cis* |

| Interval | Chr | SNP | Position (bp) | fA1 | fA2 | *p*-value | *q*-value | Associated Gene | Consequence | Ensembl_GeneId | Gene Id | *Cis/Trans*-eSNPs |
|---|---|---|---|---|---|---|---|---|---|---|---|---|
| I2 | 3 | MARC0101263 | 26425965 | 0.83 | 0.17 | $1.11x10^{-17}$ | $1.57x10^{-14}$ | *ACSM5* | intergenic | - | - | *cis* |
| I2 | 3 | MARC0050331 | 26428824 | 0.79 | 0.21 | $4.44x10^{-16}$ | $5.66x10^{-13}$ | *ACSM5* | intergenic | - | - | *cis* |
| I2 | 3 | MARC0019934 | 26488381 | 0.34 | 0.66 | $1.36x10^{-5}$ | $3.61x10^{-3}$ | *ACSM5* | intergenic | - | - | *cis* |
| I2 | 3 | MARC0007734 | 26517625 | 0.66 | 0.34 | $1.56x10^{-10}$ | $1.15x10^{-7}$ | *ACSM5* | intergenic | - | - | *cis* |
| I2 | 3 | MARC0052941 | 26537702 | 0.73 | 0.27 | $1.11x10^{-17}$ | $1.57x10^{-14}$ | *ACSM5* | downstream | ENSSSCG00000007862 | *GPR139* | *cis* |
| I2 | 3 | ALGA0018104 | 26631496 | 0.49 | 0.51 | $1.89x10^{-6}$ | $6.34x10^{-4}$ | *ACSM5* | intergenic | - | - | *cis* |
| I2 | 3 | M1GA0004189 | 26724456 | 0.40 | 0.60 | $3.53x10^{-5}$ | $8.24x10^{-3}$ | *ACSM5* | intergenic | - | - | *cis* |
| I2 | 3 | MARC0001269 | 26822485 | 0.72 | 0.28 | $1.11x10^{-17}$ | $1.57x10^{-14}$ | *ACSM5* | intergenic | - | - | *cis* |
| I2 | 3 | ALGA0106209 | 26913735 | 0.43 | 0.57 | $3.16x10^{-8}$ | $1.61x10^{-5}$ | *ACSM5* | intronic | ENSSSCG00000029212 | *GDE1* | *cis* |
| I2 | 3 | ALGA0121590 | 26978899 | 0.52 | 0.48 | $2.66x10^{-15}$ | $2.83x10^{-12}$ | *ACSM5* | intronic | ENSSSCG00000007866 | *TMC7* | *cis* |
| I2 | 3 | ASGA0090088 | 27058670 | 0.77 | 0.23 | $1.11x10^{-17}$ | $1.57x10^{-14}$ | *ACSM5* | intronic | ENSSSCG00000007868 | *TMC5* | *cis* |
| I2 | 3 | MARC0094635 | 27083041 | 0.47 | 0.53 | $1.13x10^{-6}$ | $4.12x10^{-4}$ | *ACSM5* | intergenic | - | - | *cis* |
| I2 | 3 | ASGA0105223 | 27200658 | 0.77 | 0.23 | $1.11x10^{-17}$ | $1.57x10^{-14}$ | *ACSM5* | intronic | ENSSSCG00000022200 | - | *cis* |
| I2 | 3 | ALGA0124353 | 27208947 | 0.53 | 0.47 | $9.98x10^{-6}$ | $2.75x10^{-3}$ | *ACSM5* | intronic | ENSSSCG00000022200 | - | *cis* |
| I2 | 3 | SIRI0001454 | 27254477 | 0.77 | 0.23 | $1.11x10^{-17}$ | $1.57x10^{-14}$ | *ACSM5* | intronic | ENSSSCG00000022200 | - | *cis* |
| I2 | 3 | MARC0010219 | 27473051 | 0.44 | 0.56 | $1.56x10^{-6}$ | $5.48x10^{-4}$ | *ACSM5* | intergenic | - | - | *cis* |
| I2 | 3 | ASGA0090426 | 27551099 | 0.56 | 0.44 | $2.20x10^{-8}$ | $1.17x10^{-5}$ | *ACSM5* | intergenic | - | - | *cis* |
| I2 | 3 | ALGA0018136 | 27799603 | 0.47 | 0.53 | $4.95x10^{-6}$ | $1.42x10^{-3}$ | *ACSM5* | intronic | ENSSSCG00000007872 | - | *cis* |
| I2 | 3 | ALGA0018138 | 27839633 | 0.71 | 0.29 | $1.11x10^{-17}$ | $1.57x10^{-14}$ | *ACSM5* | intronic | ENSSSCG00000007872 | - | *cis* |
| I2 | 3 | MARC0000263 | 27855477 | 0.59 | 0.41 | $3.32x10^{-7}$ | $1.32x10^{-4}$ | *ACSM5* | intronic | ENSSSCG00000007872 | - | *cis* |
| I2 | 3 | MARC0046257 | 27878993 | 0.79 | 0.21 | $1.11x10^{-17}$ | $1.57x10^{-14}$ | *ACSM5* | intronic | ENSSSCG00000007872 | - | *cis* |
| I2 | 3 | ALGA0018155 | 27912117 | 0.66 | 0.34 | $1.37x10^{-7}$ | $6.10x10^{-5}$ | *ACSM5* | upstream | ENSSSCG00000007872 | - | *cis* |
| I2 | 3 | ALGA0018160 | 27925965 | 0.66 | 0.34 | $2.36x10^{-7}$ | $9.92x10^{-5}$ | *ACSM5* | intergenic | - | - | *cis* |
| I2 | 3 | ASGA0013982 | 27941321 | 0.71 | 0.29 | $1.11x10^{-17}$ | $1.57x10^{-14}$ | *ACSM5* | intergenic | - | - | *cis* |
| I2 | 3 | ASGA0013988 | 27988181 | 0.65 | 0.35 | $1.01x10^{-5}$ | $2.76x10^{-3}$ | *ACSM5* | intergenic | - | - | *cis* |

| Interval | Chr | SNP | Position (bp) | fA1 | fA2 | *p*-value | *q*-value | Associated Gene | Consequence | Ensembl_GeneId | Gene Id | *Cis/Trans*-eSNPs |
|---|---|---|---|---|---|---|---|---|---|---|---|---|
| I2 | 3 | ALGA0018199 | 28346892 | 0.66 | 0.34 | $9.35x10^{-7}$ | $3.47x10^{-4}$ | *ACSM5* | intergenic | - | - | *cis* |
| I2 | 3 | ASGA0014014 | 28373428 | 0.65 | 0.35 | $2.85x10^{-7}$ | $1.17x10^{-4}$ | *ACSM5* | upstream | ENSSSCG00000019735 | *5S_rRNA* | *cis* |
| I2 | 3 | ASGA0014023 | 28786813 | 0.67 | 0.33 | $2.59x10^{-6}$ | $8.47x10^{-4}$ | *ACSM5* | intergenic | - | - | *cis* |
| I2 | 3 | ASGA0104499 | 28886788 | 0.54 | 0.46 | $4.94x10^{-7}$ | $1.93x10^{-4}$ | *ACSM5* | downstream | ENSSSCG00000019945 | *U6* | *cis* |
| I2 | 3 | ASGA0014039 | 29752690 | 0.61 | 0.39 | $2.89x10^{-5}$ | $7.18x10^{-3}$ | *ACSM5* | intronic | ENSSSCG00000007878 | *PARN* | *cis* |
| I2 | 3 | H3GA0009179 | 30781487 | 0.63 | 0.37 | $4.30x10^{-6}$ | $1.27x10^{-3}$ | *ACSM5* | intergenic | - | - | *cis* |
| I2 | 3 | CASI0006297 | 32293788 | 0.54 | 0.46 | $2.90x10^{-7}$ | $1.18x10^{-4}$ | *ACSM5* | intergenic | - | - | *trans* |
| I2 | 3 | M1GA0024387 | 32474314 | 0.55 | 0.45 | $6.00x10^{-8}$ | $2.83x10^{-5}$ | *ACSM5* | intergenic | - | - | *trans* |
| I2 | 3 | ALGA0119050 | 32501522 | 0.55 | 0.45 | $6.00x10^{-8}$ | $2.83x10^{-5}$ | *ACSM5* | intergenic | - | - | *trans* |
| I2 | 3 | MARC0015229 | 32509563 | 0.55 | 0.45 | $6.00x10^{-8}$ | $2.83x10^{-5}$ | *ACSM5* | upstream | ENSSSCG00000007899 | - | *trans* |
| I2 | 3 | MARC0031378 | 32543630 | 0.55 | 0.45 | $1.63x10^{-7}$ | $7.09x10^{-5}$ | *ACSM5* | intergenic | - | - | *trans* |
| I2 | 3 | MARC0004212 | 34672106 | 0.44 | 0.56 | $1.18x10^{-10}$ | $8.93x10^{-8}$ | *ACSM5* | synonymous | ENSSSCG00000007909 | *ABAT* | *trans* |
| I2 | 3 | M1GA0004280 | 34875865 | 0.62 | 0.38 | $1.60x10^{-6}$ | $5.52x10^{-4}$ | *ACSM5* | intergenic | - | - | *trans* |
| I2 | 3 | ASGA0014213 | 34921528 | 0.50 | 0.50 | $1.39x10^{-8}$ | $7.71x10^{-6}$ | *ACSM5* | intronic | ENSSSCG00000007914 | - | *trans* |
| I2 | 3 | ALGA0115191 | 34936811 | 0.30 | 0.70 | $1.16x10^{-5}$ | $3.13x10^{-3}$ | *ACSM5* | intronic | ENSSSCG00000007914 | - | *trans* |
| I2 | 3 | ALGA0107071 | 35759334 | 0.69 | 0.31 | $1.06x10^{-11}$ | $9.22x10^{-9}$ | *ACSM5* | intergenic | - | - | *trans* |
| I2 | 3 | ALGA0102473 | 37034994 | 0.76 | 0.24 | $8.97x10^{-8}$ | $4.14x10^{-5}$ | *ACSM5* | intergenic | - | - | *trans* |
| I2 | 3 | MARC0020793 | 37234505 | 0.77 | 0.23 | $5.05x10^{-7}$ | $1.95x10^{-4}$ | *ACSM5* | intergenic | - | - | *trans* |
| I2 | 3 | ASGA0105173 | 39375927 | 0.37 | 0.63 | $1.52x10^{-5}$ | $3.93x10^{-3}$ | *ACSM5* | intergenic | - | - | *trans* |
| I2 | 3 | ALGA0105074 | 39433703 | 0.37 | 0.63 | $1.52x10^{-5}$ | $3.93x10^{-3}$ | *ACSM5* | intergenic | - | - | *trans* |
| I2 | 3 | ALGA0114510 | 39644923 | 0.37 | 0.63 | $1.52x10^{-5}$ | $3.93x10^{-3}$ | *ACSM5* | intronic | ENSSSCG00000007951 | *CREBBP* | *trans* |
| I2 | 3 | H3GA0009309 | 39858459 | 0.37 | 0.63 | $1.52x10^{-5}$ | $3.93x10^{-3}$ | *ACSM5* | downstream | ENSSSCG00000007953 | *DNASE1* | *trans* |
| I2 | 3 | ALGA0018568 | 39881683 | 0.53 | 0.47 | $6.11x10^{-6}$ | $1.73x10^{-3}$ | *ACSM5* | intergenic | - | - | *trans* |
| I2 | 3 | M1GA0004302 | 39992614 | 0.53 | 0.47 | $6.11x10^{-6}$ | $1.73x10^{-3}$ | *ACSM5* | downstream | ENSSSCG00000007954 | *SLX4* | *trans* |
| I2 | 3 | ASGA0093403 | 46893143 | 0.75 | 0.25 | $7.25x10^{-11}$ | $5.66x10^{-8}$ | *ACSM5* | intergenic | - | - | *trans* |

| Interval | Chr | SNP | Position (bp) | fA1 | fA2 | *p*-value | *q*-value | Associated Gene | Consequence | Ensembl_GeneId | Gene Id | *Cis/Trans*-eSNPs |
|---|---|---|---|---|---|---|---|---|---|---|---|---|
| I2 | 3 | H3GA0009347 | 47890224 | 0.53 | 0.47 | $3.38x10^{-10}$ | $2.35x10^{-7}$ | *ACSM5* | intergenic | - | - | *trans* |
| I2 | 3 | ALGA0018674 | 47957752 | 0.69 | 0.31 | $1.63x10^{-6}$ | $5.52x10^{-4}$ | *ACSM5* | intronic | ENSSSCG00000008111 | *NPHP1* | *trans* |
| I2 | 3 | ALGA0018683 | 47979489 | 0.69 | 0.31 | $1.63x10^{-6}$ | $5.52x10^{-4}$ | *ACSM5* | intronic | ENSSSCG00000008111 | *NPHP1* | *trans* |
| I2 | 3 | H3GA0009368 | 48216866 | 0.81 | 0.19 | $3.26x10^{-5}$ | $7.75x10^{-3}$ | *ACSM5* | intergenic | - | - | *trans* |
| I2 | 3 | MARC0071874 | 48324056 | 0.86 | 0.14 | $5.07x10^{-9}$ | $2.90x10^{-6}$ | *ACSM5* | intergenic | - | - | *trans* |
| I2 | 3 | MARC0041994 | 48345565 | 0.86 | 0.14 | $2.00x10^{-9}$ | $1.23x10^{-6}$ | *ACSM5* | downstream | ENSSSCG00000022826 | - | *trans* |
| I2 | 3 | ALGA0018791 | 48500096 | 0.77 | 0.23 | $3.02x10^{-10}$ | $2.14x10^{-7}$ | *ACSM5* | upstream | ENSSSCG00000008121 | *GPAT2* | *trans* |
| I2 | 3 | ALGA0114161 | 48855083 | 0.81 | 0.19 | $3.26x10^{-5}$ | $7.75x10^{-3}$ | *ACSM5* | intronic | ENSSSCG00000008123 | *ARID5A* | *trans* |
| I2 | 3 | ASGA0104566 | 48856852 | 0.81 | 0.19 | $3.26x10^{-5}$ | $7.75x10^{-3}$ | *ACSM5* | intronic | ENSSSCG00000008123 | *ARID5A* | *trans* |
| I2 | 3 | ALGA0018800 | 49041576 | 0.81 | 0.19 | $3.26x10^{-5}$ | $7.75x10^{-3}$ | *ACSM5* | intronic | ENSSSCG00000008127 | - | *trans* |
| I2 | 3 | ASGA0014426 | 49104674 | 0.81 | 0.19 | $3.26x10^{-5}$ | $7.75x10^{-3}$ | *ACSM5* | downstream | ENSSSCG00000008129 | - | *trans* |
| I2 | 3 | ASGA0103041 | 49443426 | 0.86 | 0.14 | $2.00x10^{-9}$ | $1.23x10^{-6}$ | *ACSM5* | intergenic | - | - | *trans* |
| I2 | 3 | ASGA0090160 | 49528314 | 0.86 | 0.14 | $2.00x10^{-9}$ | $1.23x10^{-6}$ | *ACSM5* | intergenic | - | - | *trans* |
| I2 | 3 | ALGA0018859 | 49555848 | 0.78 | 0.22 | $1.80x10^{-7}$ | $7.74x10^{-5}$ | *ACSM5* | intergenic | - | - | *trans* |
| I2 | 3 | H3GA0009485 | 50180162 | 0.85 | 0.15 | $6.17x10^{-7}$ | $2.36x10^{-4}$ | *ACSM5* | intronic | ENSSSCG00000008140 | *SLC5A7* | *trans* |
| I2 | 3 | MARC0024281 | 50793814 | 0.43 | 0.57 | $4.60x10^{-6}$ | $1.33x10^{-3}$ | *ACSM5* | intergenic | - | - | *trans* |
| I2 | 3 | ASGA0014649 | 56289772 | 0.65 | 0.35 | $2.41x10^{-5}$ | $6.19x10^{-3}$ | *ACSM5* | intergenic | - | - | *trans* |
| I2 | 3 | M1GA0004378 | 56459296 | 0.62 | 0.38 | $1.97x10^{-10}$ | $1.42x10^{-7}$ | *ACSM5* | intronic | ENSSSCG00000008175 | *CHST10* | *trans* |
| I2 | 3 | M1GA0004379 | 56484282 | 0.73 | 0.27 | $2.29x10^{-6}$ | $7.55x10^{-4}$ | *ACSM5* | 3_prime_UTR_variant | ENSSSCG00000008175 | *CHST10* | *trans* |
| I2 | 3 | INRA0010621 | 56668305 | 0.86 | 0.14 | $3.41x10^{-6}$ | $1.07x10^{-3}$ | *ACSM5* | intergenic | - | - | *trans* |
| I2 | 3 | DRGA0003897 | 56709945 | 0.86 | 0.14 | $3.41x10^{-6}$ | $1.07x10^{-3}$ | *ACSM5* | intergenic | - | - | *trans* |
| I2 | 3 | ALGA0019110 | 56746317 | 0.86 | 0.14 | $3.41x10^{-6}$ | $1.07x10^{-3}$ | *ACSM5* | intergenic | - | - | *trans* |
| I2 | 3 | ASGA0014688 | 56833842 | 0.67 | 0.33 | $4.03x10^{-9}$ | $2.34x10^{-6}$ | *ACSM5* | intronic | ENSSSCG00000008177 | *AFF3* | *trans* |
| I2 | 3 | ASGA0014690 | 56857721 | 0.73 | 0.27 | $4.09x10^{-6}$ | $1.22x10^{-3}$ | *ACSM5* | intronic | ENSSSCG00000008177 | *AFF3* | *trans* |
| I2 | 3 | ALGA0019116 | 56885984 | 0.86 | 0.14 | $3.41x10^{-6}$ | $1.07x10^{-3}$ | *ACSM5* | intronic | ENSSSCG00000008177 | *AFF3* | *trans* |

| Interval | Chr | SNP | Position (bp) | fA1 | fA2 | *p*-value | *q*-value | Associated Gene | Consequence | Ensembl_GeneId | Gene Id | *Cis/Trans*-eSNPs |
|---|---|---|---|---|---|---|---|---|---|---|---|---|
| I2 | 3 | ALGA0019121 | 57129085 | 0.73 | 0.27 | $4.09 \times 10^{-6}$ | $1.22 \times 10^{-3}$ | *ACSM5* | intronic | ENSSSCG00000008177 | *AFF3* | *trans* |
| I2 | 3 | ALGA0108239 | 57170110 | 0.73 | 0.27 | $4.09 \times 10^{-6}$ | $1.22 \times 10^{-3}$ | *ACSM5* | intronic | ENSSSCG00000008177 | *AFF3* | *trans* |
| I2 | 3 | MARC0001937 | 57187517 | 0.54 | 0.46 | $1.25 \times 10^{-9}$ | $8.25 \times 10^{-7}$ | *ACSM5* | intronic | ENSSSCG00000008177 | *AFF3* | *trans* |
| I2 | 3 | MARC0034058 | 57211150 | 0.40 | 0.60 | $2.27 \times 10^{-9}$ | $1.38 \times 10^{-6}$ | *ACSM5* | intergenic | - | - | *trans* |
| I2 | 3 | ASGA0094631 | 57219021 | 0.56 | 0.44 | $1.06 \times 10^{-6}$ | $3.90 \times 10^{-4}$ | *ACSM5* | intergenic | - | - | *trans* |
| I2 | 3 | ALGA0019125 | 57431333 | 0.73 | 0.27 | $4.09 \times 10^{-6}$ | $1.22 \times 10^{-3}$ | *ACSM5* | intronic | ENSSSCG00000008179 | *REV1* | *trans* |
| I2 | 3 | ASGA0082387 | 57704441 | 0.73 | 0.27 | $4.09 \times 10^{-6}$ | $1.22 \times 10^{-3}$ | *ACSM5* | downstream | ENSSSCG00000008185 | *MITD1* | *trans* |
| I2 | 3 | ASGA0014810 | 60580301 | 0.18 | 0.82 | $3.14 \times 10^{-5}$ | $7.75 \times 10^{-3}$ | *ACSM5* | upstream | ENSSSCG00000008213 | *CD8B* | *trans* |
| I2 | 3 | MARC0017871 | 60850148 | 0.81 | 0.19 | $3.97 \times 10^{-5}$ | $9.10 \times 10^{-3}$ | *ACSM5* | downstream | ENSSSCG00000008217 | *CD8A* | *trans* |
| I2 | 3 | ASGA0014871 | 62719672 | 0.21 | 0.79 | $3.86 \times 10^{-5}$ | $8.90 \times 10^{-3}$ | *ACSM5* | intergenic | - | - | *trans* |
| I2 | 3 | ALGA0111911 | 63248078 | 0.19 | 0.81 | $3.42 \times 10^{-5}$ | $8.03 \times 10^{-3}$ | *ACSM5* | intergenic | - | - | *trans* |
| I3 | 3 | MARC0032158 | 83365626 | 0.35 | 0.65 | $1.63 \times 10^{-6}$ | $5.52 \times 10^{-4}$ | *ACSM5* | intergenic | - | - | *trans* |
| I4 | 3 | ALGA0020206 | 100905327 | 0.70 | 0.30 | $9.34 \times 10^{-6}$ | $2.59 \times 10^{-3}$ | *ACSM5* | intergenic | - | - | *trans* |
| I4 | 3 | ASGA0015465 | 101222209 | 0.68 | 0.32 | $1.41 \times 10^{-6}$ | $5.09 \times 10^{-4}$ | *ACSM5* | intergenic | - | - | *trans* |
| I4 | 3 | ASGA0098441 | 101461883 | 0.70 | 0.30 | $7.63 \times 10^{-6}$ | $2.15 \times 10^{-3}$ | *ACSM5* | intergenic | - | - | *trans* |
| I5 | 10 | H3GA0055101 | 54839 | 0.47 | 0.53 | $8.11 \times 10^{-7}$ | $3.04 \times 10^{-4}$ | *ACSM5* | intronic | ENSSSCG00000010795 | *NDUFAB1* | *trans* |
| I5 | 10 | MARC0015344 | 65922 | 0.47 | 0.53 | $8.11 \times 10^{-7}$ | $3.04 \times 10^{-4}$ | *ACSM5* | downstream | ENSSSCG00000010795 | *NDUFAB1* | *trans* |
| I5 | 10 | ASGA0082591 | 120016 | 0.74 | 0.26 | $2.00 \times 10^{-8}$ | $1.08 \times 10^{-5}$ | *ACSM5* | downstream | ENSSSCG00000010797 | *EARS2* | *trans* |
| I5 | 10 | ASGA0092761 | 150115 | 0.65 | 0.35 | $2.78 \times 10^{-9}$ | $1.66 \times 10^{-6}$ | *ACSM5* | intronic | ENSSSCG00000010798 | *GGA2* | *trans* |
| I5 | 10 | H3GA0055120 | 167126 | 0.70 | 0.30 | $2.89 \times 10^{-15}$ | $2.99 \times 10^{-12}$ | *ACSM5* | downstream | ENSSSCG00000010798 | *GGA2* | *trans* |
| I5 | 10 | ASGA0090778 | 175359 | 0.76 | 0.24 | $1.44 \times 10^{-15}$ | $1.72 \times 10^{-12}$ | *ACSM5* | intronic | ENSSSCG00000010799 | *COG7* | *trans* |
| I5 | 10 | ASGA0094144 | 184863 | 0.71 | 0.29 | $1.09 \times 10^{-8}$ | $6.13 \times 10^{-6}$ | *ACSM5* | intronic | ENSSSCG00000010799 | *COG7* | *trans* |
| I5 | 10 | ASGA0095156 | 202837 | 0.74 | 0.26 | $2.00 \times 10^{-8}$ | $1.08 \times 10^{-5}$ | *ACSM5* | intronic | ENSSSCG00000010799 | *COG7* | *trans* |
| I6 | 16 | ALGA0089402 | 19133382 | 0.66 | 0.34 | $3.83 \times 10^{-5}$ | $8.88 \times 10^{-3}$ | *ACSM5* | intronic | ENSSSCG00000016810 | *PDZD2* | *trans* |
| I7 | 13 | ASGA0055780 | 6891435 | 0.55 | 0.45 | $1.20 \times 10^{-7}$ | $4.74 \times 10^{-3}$ | *ELOVL6* | intergenic | - | - | *trans* |

| Interval | Chr | SNP | Position (bp) | fA1 | fA2 | *p*-value | *q*-value | Associated Gene | Consequence | Ensembl_GeneId | Gene Id | *Cis/Trans*-eSNPs |
|---|---|---|---|---|---|---|---|---|---|---|---|---|
| I8 | 2 | ASGA0008719 | 6094116 | 0.48 | 0.52 | $1.26x10^{-5}$ | $4.75x10^{-2}$ | *FABP4* | intergenic | - | - | *trans* |
| I8 | 2 | MARC0018949 | 8997005 | 0.59 | 0.41 | $1.38x10^{-5}$ | $4.75x10^{-2}$ | *FABP4* | upstream | ENSSSCG00000023090 | *BEST1* | *trans* |
| I9 | 3 | ASGA0015643 | 109404009 | 0.69 | 0.31 | $1.44x10^{-5}$ | $4.75x10^{-2}$ | *FABP4* | 3_prime_UTR_variant | ENSSSCG00000020689 | *CEBPZOS* | *trans* |
| I9 | 3 | ALGA0020658 | 113469226 | 0.91 | 0.09 | $6.70x10^{-6}$ | $4.75x10^{-2}$ | *FABP4* | intronic | ENSSSCG00000008510 | *LTBP1* | *trans* |
| I9 | 3 | ASGA0016181 | 119739502 | 0.66 | 0.34 | $8.31x10^{-6}$ | $4.75x10^{-2}$ | *FABP4* | synonymous | ENSSSCG00000008568 | *DRC1* | *trans* |
| I10 | 4 | ALGA0024527 | 36727180 | 0.57 | 0.43 | $1.11x10^{-5}$ | $4.75x10^{-2}$ | *FABP4* | intergenic | - | - | *trans* |
| I11 | 4 | ALGA0025158 | 60566866 | 0.48 | 0.52 | $1.93x10^{-6}$ | $1.95x10^{-2}$ | *FABP4* | intergenic | - | - | *cis (FABP5)* |
| I11 | 4 | ALGA0025162 | 60844160 | 0.68 | 0.32 | $1.32x10^{-5}$ | $4.75x10^{-2}$ | *FABP4* | intergenic | - | - | *cis (FABP5)* |
| I11 | 4 | MARC0115316 | 61059205 | 0.52 | 0.48 | $1.97x10^{-6}$ | $1.95x10^{-2}$ | *FABP4* | intronic | ENSSSCG00000022989 | *ZNF704* | *cis (FABP5)* |
| I11 | 4 | INRA0014244 | 63090979 | 0.59 | 0.41 | $1.19x10^{-5}$ | $4.75x10^{-2}$ | *FABP4* | intronic | ENSSSCG00000006163 | PKIA | *cis (FABP5)* |
| I11 | 4 | ALGA0025337 | 65252780 | 0.77 | 0.23 | $8.67x10^{-7}$ | $1.95x10^{-2}$ | *FABP4* | intergenic | - | - | *cis (FABP5)* |
| I12 | 9 | ALGA0054847 | 129178729 | 0.88 | 0.12 | $1.19x10^{-6}$ | $1.95x10^{-2}$ | *FABP4* | intronic | ENSSSCG00000015508 | *TNR* | *trans* |
| I13 | 2 | ASGA0008845 | 7850065 | 0.61 | 0.39 | $9.88x10^{-6}$ | $3.93x10^{-2}$ | *FADS2* | intronic | ENSSSCG00000013056 | *LGALS12* | *trans* |
| I13 | 2 | ASGA0104083 | 8867970 | 0.78 | 0.22 | $5.98x10^{-8}$ | $5.94x10^{-4}$ | *FADS2* | intronic | ENSSSCG00000013066 | - | *cis* |
| I13 | 2 | ASGA0090054 | 8943379 | 0.86 | 0.14 | $2.03x10^{-6}$ | $1.15x10^{-2}$ | *FADS2* | intergenic | - | - | *cis* |
| I13 | 2 | ALGA0011764 | 9098547 | 0.89 | 0.11 | $1.54x10^{-5}$ | $4.71x10^{-2}$ | *FADS2* | intergenic | - | - | *cis* |
| I13 | 2 | ASGA0008884 | 9139348 | 0.92 | 0.08 | $1.54x10^{-5}$ | $4.71x10^{-2}$ | *FADS2* | intronic | ENSSSCG00000013072 | *FADS2* | *cis* |
| I13 | 2 | ASGA0008896 | 9171685 | 0.66 | 0.34 | $2.58x10^{-7}$ | $1.71x10^{-3}$ | *FADS2* | intronic | ENSSSCG00000013073 | *FADS3* | *cis* |
| I13 | 2 | ALGA0011760 | 9198141 | 0.86 | 0.14 | $1.21x10^{-5}$ | $4.37x10^{-2}$ | *FADS2* | upstream | ENSSSCG00000013074 | *RAB3IL1* | *cis* |
| I13 | 2 | ASGA0008874 | 9218889 | 0.79 | 0.21 | $2.28x10^{-7}$ | $1.71x10^{-3}$ | *FADS2* | intergenic | - | - | *cis* |
| I14 | 6 | ALGA0035721 | 74418977 | 0.74 | 0.26 | $7.50x10^{-6}$ | $3.31x10^{-2}$ | *FADS2* | intergenic | - | - | *trans* |
| I15 | 8 | ASGA0100508 | 59002816 | 0.35 | 0.65 | $1.93x10^{-5}$ | $4.80x10^{-2}$ | *FADS2* | intronic | ENSSSCG00000025792 | - | *trans* |
| I15 | 8 | MARC0041089 | 59080961 | 0.65 | 0.35 | $1.93x10^{-5}$ | $4.80x10^{-2}$ | *FADS2* | intronic | ENSSSCG00000008913 | *IGFBP7* | *trans* |
| I15 | 8 | H3GA0024926 | 68676342 | 0.65 | 0.35 | $1.93x10^{-5}$ | $4.80x10^{-2}$ | *FADS2* | intergenic | - | - | *trans* |
| I16 | 10 | H3GA0030086 | 46933533 | 0.66 | 0.34 | $2.40x10^{-6}$ | $1.19x10^{-2}$ | *FADS2* | intergenic | - | - | *trans* |

| Interval | Chr | SNP | Position (bp) | fA1 | fA2 | *p*-value | *q*-value | Associated Gene | Consequence | Ensembl_GeneId | Gene Id | *Cis/Trans*-eSNPs |
|---|---|---|---|---|---|---|---|---|---|---|---|---|
| I17 | 9 | MARC0034587 | 20183865 | 0.76 | 0.24 | $2.15x10^{-5}$ | $3.42x10^{-2}$ | *SLC27A4* | intergenic | - | - | *trans* |
| I17 | 9 | ASGA0041925 | 20196539 | 0.79 | 0.21 | $1.69x10^{-5}$ | $2.92x10^{-2}$ | *SLC27A4* | intergenic | - | - | *trans* |
| I18 | 14 | ASGA0064787 | 88898693 | 0.95 | 0.05 | $2.51x10^{-6}$ | $4.76x10^{-3}$ | *SLC27A4* | intergenic | - | - | *trans* |
| I18 | 14 | MARC0057510 | 89058255 | 0.95 | 0.05 | $2.51x10^{-6}$ | $4.76x10^{-3}$ | *SLC27A4* | intronic | ENSSSCG00000010342 | *SH2D4B* | *trans* |
| I18 | 14 | MARC0029597 | 89073094 | 0.95 | 0.05 | $2.51x10^{-6}$ | $4.76x10^{-3}$ | *SLC27A4* | downstream | ENSSSCG00000010342 | *SH2D4B* | *trans* |
| I18 | 14 | ALGA0079330 | 89521045 | 0.95 | 0.05 | $2.51x10^{-6}$ | $4.76x10^{-3}$ | *SLC27A4* | intergenic | - | - | *trans* |
| I18 | 14 | MARC0041088 | 90286262 | 0.95 | 0.05 | $2.51x10^{-6}$ | $4.76x10^{-3}$ | *SLC27A4* | intergenic | - | - | *trans* |
| I18 | 14 | ASGA0064841 | 90338701 | 0.95 | 0.05 | $2.51x10^{-6}$ | $4.76x10^{-3}$ | *SLC27A4* | intergenic | - | - | *trans* |
| I18 | 14 | ASGA0064844 | 90362764 | 0.95 | 0.05 | $2.51x10^{-6}$ | $4.76x10^{-3}$ | *SLC27A4* | intergenic | - | - | *trans* |
| I18 | 14 | MARC0088303 | 90797721 | 0.95 | 0.05 | $2.51x10^{-6}$ | $4.76x10^{-3}$ | *SLC27A4* | intergenic | - | - | *trans* |
| I18 | 14 | ALGA0079375 | 90925232 | 0.95 | 0.05 | $2.51x10^{-6}$ | $4.76x10^{-3}$ | *SLC27A4* | intergenic | - | - | *trans* |
| I18 | 14 | MARC0010015 | 91126881 | 0.95 | 0.05 | $2.51x10^{-6}$ | $4.76x10^{-3}$ | *SLC27A4* | intergenic | - | - | *trans* |
| I18 | 14 | MARC0003938 | 91360798 | 0.91 | 0.09 | $6.84x10^{-6}$ | $1.24x10^{-2}$ | *SLC27A4* | intergenic | - | - | *trans* |
| I18 | 14 | ALGA0079393 | 91666877 | 0.95 | 0.05 | $2.51x10^{-6}$ | $4.76x10^{-3}$ | *SLC27A4* | intergenic | - | - | *trans* |
| I18 | 14 | MARC0056155 | 91685430 | 0.95 | 0.05 | $2.51x10^{-6}$ | $4.76x10^{-3}$ | *SLC27A4* | intergenic | - | - | *trans* |
| I18 | 14 | ALGA0079399 | 92014581 | 0.95 | 0.05 | $2.51x10^{-6}$ | $4.76x10^{-3}$ | *SLC27A4* | intergenic | - | - | *trans* |
| I18 | 14 | MARC0014799 | 92290727 | 0.95 | 0.05 | $2.51x10^{-6}$ | $4.76x10^{-3}$ | *SLC27A4* | intronic | ENSSSCG00000010350 | *RGR* | *trans* |
| I18 | 14 | ALGA0079407 | 92432834 | 0.95 | 0.05 | $2.51x10^{-6}$ | $4.76x10^{-3}$ | *SLC27A4* | intronic | ENSSSCG00000010351 | *CCSER2* | *trans* |
| I19 | 15 | ASGA0070790 | 137054337 | 0.93 | 0.07 | $3.39x10^{-5}$ | $5.00x10^{-2}$ | *SLC27A4* | intergenic | - | - | *trans* |
| I19 | 15 | MARC0050960 | 137365338 | 0.93 | 0.07 | $3.39x10^{-5}$ | $5.00x10^{-2}$ | *SLC27A4* | intergenic | - | - | *trans* |

**Table S3.** Transcription factor binding sites for *PPARG* in the *ACSM5* promoter region gene.

| TF Name | Sequence | Position* | Strand | Score | P-value |
|---|---|---|---|---|---|
| PPARG(M00512) | TTTGGGGCCAGAGGTC | 3:26,260,381-26,260,365 | + | 10.16 | $2.50x10^{-4}$ |
| PPARG(M00512) | CTCAGCGTCAAAGGCAA | 3:26,260,939-26,260,922 | - | 6.66 | $7.50x10^{-4}$ |

* Chromosomal position is given according to *Sscrofa10.2* assembly coordinates

**Table S4.** Transcription factor binding site for *SREBF1* in the *FADS2* promoter region gene.

| TF Name | Sequence | Position* | Strand | Score | P-value |
|---|---|---|---|---|---|
| SREBF1(M00220) | CATCAGGTG | 2:9,118,880-9,118,889 | + | 9.23 | $5.50 \times 10^{-4}$ |

* Chromosomal position is given according to *Sscrofa10.2* assembly coordinates

**Table S6.** Mean comparison between males and females of backfat gene expression levels of 43 lipid-related genes.

| Gene | Mean in Male (SEM[a]) | Mean in Female (SEM[a]) | *P*-value |
|---|---|---|---|
| *ACSM5* | 0.5182 (0.1303) | 1.7285 (0.2489) | $3.13x10^{-4}$ |
| *ADIPOQ* | 0.9216 (0.0444) | 0.9828 (0.0290) | $2.36x10^{-1}$ |
| *AGPAT2* | 0.8792 (0.0478) | 1.1046 (0.0467) | $1.37x10^{-3}$ |
| *ANK2* | 0.8480 (0.0357) | 1.1495 (0.0379) | $2.29x10^{-7}$ |
| *ARNT* | 1.0924 (0.0393) | 1.1204 (0.0227) | $5.19x10^{-1}$ |
| *CD36* | 0.9962 (0.0380) | 0.9262 (0.0255) | $1.19x10^{-1}$ |
| *CPT1A* | 0.9173 (0.0451) | 1.0113 (0.0372) | $1.12x10^{-1}$ |
| *CROT* | 0.9526 (0.0487) | 1.0587 (0.0456) | $1.22x10^{-1}$ |
| *CYP2U1* | 1.0761 (0.0621) | 1.1121 (0.0403) | $6.16x10^{-1}$ |
| *DGAT1* | 1.0078 (0.0818) | 1.1463 (0.0575) | $1.60x10^{-1}$ |
| *DGAT2* | 0.8239 (0.0852) | 1.2077 (0.1066) | $9.35x10^{-3}$ |
| *EGF* | 0.6850 (0.0719) | 1.7432 (0.5234) | $8.85x10^{-2}$ |
| *ELOVL5* | 0.9596 (0.0657) | 1.1672 (0.0642) | $2.98x10^{-2}$ |
| *ELOVL6* | 0.7408 (0.0769) | 1.2435 (0.1106) | $7.89x10^{-4}$ |
| *ESRRA* | 1.0523 (0.0441) | 1.1324 (0.0547) | $2.85x10^{-1}$ |
| *FABP4* | 0.9172 (0.0298) | 0.9783 (0.0361) | $2.20x10^{-1}$ |
| *FABP5* | 0.8709 (0.0395) | 0.8328 (0.0288) | $4.29x10^{-1}$ |
| *FADS1* | 0.8151 (0.0421) | 0.8192 (0.0286) | $9.35x10^{-1}$ |
| *FADS2* | 1.0317 (0.0630) | 0.8606 (0.0388) | $1.79x10^{-2}$ |
| *FADS3* | 0.9534 (0.0321) | 0.9414 (0.0208) | $7.46x10^{-1}$ |
| *LIPC* | 1.0681 (0.0678) | 1.0736 (0.0429) | $9.4410^{-1}$ |
| *LPIN1* | 0.9895 (0.0544) | 0.9046 (0.0408) | $2.09x10^{-1}$ |
| *MGLL* | 0.9194 (0.0456) | 1.0508 (0.0376) | $2.84x10^{-2}$ |
| *MLXIPL* | 0.9647 (0.0625) | 1.2457 (0.0582) | $1.65x10^{-3}$ |
| *NFKB1* | 0.9855 (0.0256) | 0.8969 (0.0177) | $4.24x10^{-3}$ |
| *NR1H3* | 1.0563 (0.0473) | 1.0664 (0.0333) | $8.59x10^{-1}$ |
| *PEX2* | 0.8274 (0.0523) | 1.1094 (0.0437) | $7.24x10^{-5}$ |
| *PLA2G12A* | 0.8832 (0.0521) | 1.1725 (0.0484) | $1.19x10^{-4}$ |
| *PLCB2* | 0.9450 (0.0411) | 0.9023 (0.0420) | $4.85x10^{-1}$ |
| *PLPP1* | 1.0787 (0.0416) | 0.8176 (0.0311) | $1.47x10^{-6}$ |
| *PNPLA2* | 0.9063 (0.0411) | 1.0913 (0.0365) | $1.21x10^{-3}$ |
| *POU2F1* | 0.9196 (0.0249) | 0.9700 (0.0264) | $1.84x10^{-1}$ |
| *PPARA* | 0.8984 (0.0652) | 1.2135 (0.0510) | $2.13x10^{-4}$ |
| *PPARD* | 0.8802 (0.0451) | 1.2035 (0.0500) | $1.15x10^{-5}$ |
| *PPARG* | 0.9723 (0.0460) | 1.0565 (0.0362) | $1.51x10^{-1}$ |
| *RBP4* | 1.1959 (0.0784) | 1.2042 (0.0667) | $9.36x10^{-1}$ |
| *RXRG* | 1.1373 (0.0804) | 1.1111 (0.0849) | $8.30x10^{-1}$ |
| *SCAP* | 0.9765 (0.0389) | 1.1847 (0.0322) | $7.55x10^{-5}$ |
| *SCD* | 0.4763 (0.6976) | 1.0685 (0.1701) | $7.40x10^{-3}$ |
| *SLC27A1* | 1.1737 (0.0694) | 1.0792 (0.0504) | $2.65x10^{-1}$ |
| *SLC27A4* | 1.1798 (0.0452) | 1.1247 (0.0314) | $3.09x10^{-1}$ |
| *SREBF1* | 0.8074 (0.0516) | 1.2552 (0.0612) | $5.86x10^{-7}$ |
| *USF1* | 1.0324 (0.0362) | 1.0333 (0.0191) | $9.82x10^{-1}$ |

[a]Standard error of the mean (SEM)

**Table S7.** Description of the 393 human orthologous genes.

| Interval | Human associated gene name |
|---|---|
| I1 | *ALDH7A1, C5orf63, CTXN3, LMNB1, MARCH3, MEGF10, PHAX, PRRC1, TEX43, GRAMD3* |
| I2 | *ACSM5* |
| I3 | *B3GNT2, CCT4, COMMD1, EHBP1, FAM161A, NUP54, OTX1, USP34, XPO1, MDH1* |
| I4 | *ATP6V1E2, CAMKMT, EPAS1, PPM1B, PREPL, PRKCE, RHOQ, SIX2, SLC3A1, SRBD1, TMEM247, PIGF* |
| I5 | *COG7, EARS2, GGA2, NDUFAB1, PALB2, UBFD1, DCTN* |
| I6 | *C5orf22, DROSHA, PDZD2, CDH6* |
| I7 | *EFHB, KCNH8, SATB1* |
| I8 | *AHNAK, AP000721.4, AP5B1, ARL2, ASRGL1, ATG2A, ATL3, B3GAT3, B4GAT1, BAD, BANF1, BATF2, BEST1, BRMS1, BSCL2, C11orf68, C11orf84, C11orf95, C11orf98, CAPN1, CATSPER1, CCDC85B, CCDC88B, CD248, CDC42BPG, CDC42EP2, CDCA5, CFL1, CHRM1, CNIH2, CPSF7, CST6, CTSW, CYB561A3, DAGLA, DDB1, DNAJC4, DPF2, DRAP1, EEF1G, EFEMP2, EHD1, EIF1AD, EML3, ESRRA, FADS1, FADS2, FADS3, FAU, FEN1, FERMT3, FIBP, FKBP2, FLRT1, FOSL1, FRMD8, GAL3ST3, GANAB, GPHA2, GPR137, HNRNPUL2, HRASLS5, INCENP, INTS5, KAT5, KCNK4, KCNK7, KLC2, LGALS12, LRRC10B, LTBP3, MACROD1, MAP4K2, MARK2, MEN1, METTL12, MRPL49, MTA2, MUS81, MYRF, NAA40, NAALADL1, NRXN2, NUDT22, NXF1, OTUB1, OVOL1, PACS1, PCNXL3, PGA4, PLA2G16, PLCB3, POLA2, POLR2G, PPP1R14B, PPP1R32, PPP2R5B, PRDX5, PYGM, RAB3IL1, RASGRP2, RCOR2, RELA, RIN1, RNASEH2C, ROM1, RPL13A, RPL22, RPS6KA4, RTN3, SAC3D1, SART1, SCGB1A1, SCGB1D2, SCYL1, SDHAF2, SF1, SF3B2, SIPA1, SLC22A10, SLC22A11, SLC22A12, SLC22A6, SLC22A8, SLC25A45, SLC3A2, SNX15, SNX32, SSSCA1, STIP1, STX5, SYT7, SYVN1, TAF6L, TEX40, TIGD3, TKFC, TM7SF2, TMEM138, TMEM151A, TMEM216, TMEM223, TMEM258, TMEM262, TRMT112, TRPT1, TSGA10IP, TTC9C, TUT1, UBXN1, UQCC3, VEGFB, VPS51, VWCE, WDR74, YIF1A, ZBTB3, ZFPL1, ZNHIT2, SCGB2A1, SLC29A2* |
| I9 | *ABHD1, ADGRF3, AGBL5, ALK, ASXL2, ATRAID, BIRC6, BRE, C2orf16, C2orf70, C2orf71, CAPN14, CCDC121, CDC42EP3, CEBPZ, CEBPZOS, CENPA, CGREF1, CIB4, CLIP4, CRIM1, DNMT3A, DPY30, DPYSL5, DRC1, DTNB, EHD3, EIF2B4, EMILIN1, EPT1, FAM179A, FAM98A, FEZ2, FNDC4, FOSL2, GAREM2, GCKR, GPATCH11, GPN1, HADHA, HADHB, HEATR5B, IFT172, KHK, KIF3C, KRTCAP3, LBH, LCLAT1, LTBP1, MAPRE3, MEMO1, NDUFAF7, NLRC4, NRBP1, PLB1, PPM1G, PPP1CB, PREB, PRKD3, PRR30, QPCT, RAB10, RASGRP3, RBKS, RMDN2, SLC30A6, SLC35F6, SLC4A1AP, SLC5A6, SNX17, SPAST, SPDYA, SRD5A2, STRN, SUPT7L, TCF23, TMEM214, TRMT61B, TTC27, VIT, WDR43, XDH, YPEL5, YWHAE, ZNF512, CYP1B1, GALNT14* |
| I10 | *ATP6V1C1, AZIN1, BAALC, CTHRC1, DCAF13, FZD6, KLF10, ODF1, RRM2B, SLC25A32, UBR5, RIMS2* |
| I11 | *FABP5* |
| I12 | *CACYBP, GPR52, KIAA0040, MRPS14, PAPPA2, RABGAP1L, RFWD2, TNN, TNR, NXPE2* |
| I14 | *C1orf234, C1QA, C1QB, C1QC, CDC42, CNR2, ECE1, EPHA8, EPHB2, FUCA1, GALE, HMGCL, HSPG2, HTR1D, KDM1A, LACTBL1, LUZP1, ZBTB40* |
| I15 | *ADGRL3, CENPC, CEP135, CLOCK, EPHA5, EXOC1, IGFBP7, NOA1, PDCL2, POLR2B, PPIC, REST, TECRL, TMEM165, TMSB4X* |
| I16 | *ARHGAP12, CUBN, EPC1, ERV3-1, KIF5B, PTCHD3, SVIL, ZEB1, ZNF438* |
| I17 | *ANKRD42, CCDC90B, DLG2, PCF11, PRCP, RAB30* |
| I18 | *ANXA11, C10orf99, CCSER2, CDHR1, DYDC1, DYDC2, FAM213A, GHITM, GRID1, LRIT1, LRIT2, MAT1A, NRG3, PLAC9, PPIF, RGR, SFTPA1, SFTPD, SH2D4B, TMEM254, TSPAN14, ZMIZ1* |
| I19 | *EPHA4, FARSB, MOGAT1, PAX3, SGPP2* |

**Table S8.** Top functional networks and molecules identified with IPA form the list of annotated genes mapping within the 19 eQTLs.

| ID | Interval | Top Diseases and Functions | Score | Focus Molecules | Molecules in Network |
|---|---|---|---|---|---|
| 1 | I1 | Cell-To-Cell Signaling and Interaction, Hematological System Development and Function, Immune Cell Trafficking | 21 | 7 | ALDH4A1,ALDH7A1,ALDH8A1,APBB3,APP,ARHGAP24,C3orf62,C9orf78,CASC4,CCL26,CREBBP,CXCL12,DPY19L3,EVI2A,Fcr,FRMD8,GIMAP4,GIMAP5,GIMAP8,GPR85,GRAMD3,GRAMD1C,LMNB1,MARCH3,MEGF10,NMNAT2,Oas1b,PHAX,PRRC1,RGL1,RTP4,SYK,TMOD4,VAV1,ZCCHC12 |
| 2 | I3 | Carbohydrate Metabolism, Organ Morphology, Reproductive System Development and Function | 30 | 10 | B3GNT2,B3GNT7,B4GAT1,BRCA1,C16orf62,CCT4,COMMD1,DBR1,EHBP1,EXD2,FAM161A,FAM173A,FAM83A,GAREM1,Hd-neuronal intranuclear inclusions,HEATR5B,HTT,LSM14B,MAGEB18,MDH1,MFAP4,NTRK1,NUP54,OTX1,PLEKHA6,POU5F1,PRRT4,Ptprv,RWDD4,TP53,USP29,USP34,WDR91,XPO1,ZC3H7A |
| 3 | I4 | Connective Tissue Disorders, Organismal Injury and Abnormalities, Cell Death and Survival | 29 | 10 | ALKBH7,APP,C11orf52,C11orf71,C2orf49,C9orf64,CAMKMT,CD40LG,CYB561D2,DTWD1,EPAS1,HNF4A,HPF1,Insulin,MCEE,MPV17L,NBPF3,NFkB (complex),NTRK1,omega-muricholic acid,PI3K (complex),PIGF,PPIP5K2,PPM1B,PREPL,PRKCE,PURG,RHOQ,RSPH3,SIX2,SLC3A1,SRBD1,THYN1,TP53,TP53TG5 |
| 4 | I4 | Molecular Transport, Hereditary Disorder, Metabolic Disease | 3 | 1 | ATP6V1B1,ATP6V1C2,ATP6V1E2,ATP6V1G1,C9orf16,H+-exporting ATPase,RICTOR,Vacuolar H+ ATPase |
| 5 | I5 | Cancer, Organismal Injury and Abnormalities, Reproductive System Disease | 21 | 7 | ACOT1,AIM1,ANGPTL2,CDC42SE1,CHST2,CNNM4,COG7,CUL3,DCTN5,DDX10,EARS2,ERBB2,ESR1,FAM98B,GGA2,LCN1,MAN1A1,NDUFAB1,NTRK1,PALB2,PCDH8,PCNA,PGM3,PRR15L,PTPRT,RHOBTB2,RTN4RL1,SPAG1,SPAG4,SUPV3L1,THADA,UBAP2,UBFD1,WDR81,ZSWIM8 |
| 6 | I6 | Cellular Development, Embryonic Development, Hair and Skin Development and Function | 12 | 4 | BCL9,Bcl9-Cbp/p300-Ctnnb1-Lef/Tcf,C12orf49,C5orf22,CASC4,CDH6,CDH7,CDH8,CDH9,CDH10,CDH15,CDH18,CNMD,CTBS,CTDSPL,CTNNB1,CTNNβ-LEF1,DACT3,DROSHA,DUSP7,ELAVL1,FBXO8,GLIPR1,GPX2,MUM1,MYBPH,MYC,PDZD2,RPL41,TCF4-CTNNβ,TMEM2,Trp53cor1,UST,VEZT,ZXDB |
| 7 | I7 | Immunological Disease, Inflammatory Disease, Inflammatory Response | 4 | 1 | IL1B,KCNH8,PTGER3 |
| 8 | I7 | Cell Cycle, Organ Morphology, Visual System Development and Function | 3 | 1 | ABTB1,BTLA,CLEC2B,CORO6,Cux1,ENOSF1,EPSTI1,EVI2A,FAM110A,FAM129A,FAM65B,FOXJ3,Foxp2,GPR18,GPT2,HELZ2,HLA-DOB,HSF5,HVCN1,Ighen,LAG3,LPIN2,LRRN3,MT1A,MYCL,PIK3IP1,POU3F4,QSER1,SATB1,TBX6,TMEM2,TMEM117,TMEM241,ZKSCAN8,ZNF287 |

| ID | Interval | Top Diseases and Functions | Score | Focus Molecules | Molecules in Network |
|---|---|---|---|---|---|
| 9 | I8 | Cell Cycle, DNA Replication, Recombination, and Repair, Developmental Disorder | 52 | 26 | 26sProteasome,Actin,BANF1,CAPN1,caspase,CD3,CTSW,DDB1,DPF2,EEF1G,ESRRA,FEN1,FERMT3,FKBP2,HISTONE,Histone h3,Histone h4,INCENP,INTS5,KAT5,KCNK4,MAP4K2,MTA2,MUS81,NFkB (complex),OTUB1,POLA2,POLR2G,PRDX5,RNA polymerase II,RPL13A,SF1,SF3B2,TAF6L,UBXN1 |
| 10 | I8 | Lipid Metabolism, Small Molecule Biochemistry, Infectious Diseases | 42 | 22 | Akt,BSCL2,Calmodulin,CDCA5,CHRM1,Ck2,ERK,estrogen receptor,FADS1,FADS2,FAU,FIBP,FSH,GANAB,Hsp90,Insulin,LGALS12,Lh,Mapk,MYRF,PLA2,PLA2G16,PLC,PLCB3,PPP2R5B,RELA,SCGB1A1,SLC22A8,SLC3A2,STAT5a/b,STIP1,STX5,SYT7,SYVN1,TMEM216 |
| 11 | I8 | Lipid Metabolism, Small Molecule Biochemistry, Cellular Assembly and Organization | 32 | 18 | AES,AHNAK,ARL2,C11orf68,C20orf194,CCT3,CDC37,CPSF7,DAGLA,DUS3L,EIF1AD,EML3,GPR137,GPR174,HSP90AA1,KCNK7,KLF3,MAP3K1,MAP3K15,MRPL49,MYLK4,NUDT22,NXF1,PDIK1L,PSKH1,RAB3IL1,SSSCA1,STAT4,SUPT5H,TRMT112,TTC9C,TUBB,TUT1,YWHAQ,ZBTB3 |
| 12 | I8 | Cellular Assembly and Organization, Cell-To-Cell Signaling and Interaction, Nervous System Development and Function | 25 | 15 | 14-3-3,Alp,Ap1,BAD,BRMS1,CDC42EP2,CFL1,Creb,ERK1/2,F Actin,Fibrinogen,FOSL1,Igg3,IgG2a,Igm,Immunoglobulin,KLC2,LDL,LTBP3,MARK2,MEN1,PACS1,PDGF BB,Pka,Pka catalytic subunit,PP2A,Rap1,Ras,RASGRP2,RIN1,Rock,RPS6KA4,Rsk,SIPA1,VEGFB |
| 13 | I8 | Cellular Compromise, Gastrointestinal Disease, Hepatic System Disease | 25 | 15 | ABHD2,ATG2A,BRCA1,C11orf84,C11orf98,CDC42BPG,Cyp2d1/Cyp2d5,DNAJB1,Dnajb1-Hsp70,DNAJC4,DNAJC16,EGFR,EHD1,Esr1-Estrogen-Sp1,FAM153A/FAM153B,HLA-C,HNRNPUL2,HSCB,HSPA9,HSPB1,lipid,MACROD1,MDC1,NAA40,PGA5 (includes others),RCOR2,RPL22,SDHAF2,SP1,SUMO2,TMCO3,TMEM223,tretinoin,TSGA10IP,XRCC5 |
| 14 | I8 | Nutritional Disease, Cell Morphology, Cell-To-Cell Signaling and Interaction | 21 | 13 | APP,ATL3,C15orf39,CACNG2,CACNG3,CACNG8,CNIH2,DRAP1,FRMD8,GORAB,GRIA1,HIST1H2AD,HRASLS5,INIP,MAD2L1,N-acetyl-L-aspartic acid,OLFM1,OLFM3,PDCD7,PPP1R32,RABL3,REEP6,RTN3,SAC3D1,SCYL1,SLC22A6,SLC30A3,SNX1,SNX14,SNX15,SNX32,SP140L,TCP11L1,TRPT1,ZCCHC6 |
| 15 | I8 | Cancer, Cell Death and Survival, Organismal Injury and Abnormalities | 21 | 13 | ADIRF,AP5B1,ASRGL1,B3GAT3,BCL2,beta-estradiol,CAMK2B,CD248,FADS3,GSK3B,IFNB1,JUNB,PELP1,PHACTR3,PP1 protein complex group,PPP1R11,PPP1R17,PPP1R14B,PPP1R14C,PPP1R14D,PPP2CA,Ppp2r3d,PSMC3,PXYLP1,PYGM,REM1,RNASEH2C,SCGB2A1,TGFB2,TKFC,TMEM44,TMEM258,VPS51,WDR74,YAP/TAZ |
| 16 | I8 | Cellular Function and Maintenance, Organ Development, Reproductive System Development and Function | 19 | 12 | adenosine,AIFM2,CACNA1I,CATSPER,CATSPER1,CATSPER2,CATSPER3,CATSPER4,CYB561A3,EFEMP2,EGF,KCTD11,LSM14A,NRXN2,PPARD,PRPF8,RNU4-1,RNU5A-1,ROM1,SART1,SLC29A2,SLC30A6,SNRNP27,TMED7,TMEM17,TMEM138,TMEM151A,TP53,UBIAD1,UBL5,USO1,voltage-gated calcium channel,YIF1A,ZFPL1,ZNHIT2 |

| ID | Interval | Top Diseases and Functions | Score | Focus Molecules | Molecules in Network |
|---|---|---|---|---|---|
| 17 | I8 | Cell Death and Survival, Gastrointestinal Disease, Organismal Injury and Abnormalities | 17 | 11 | ADGRA1,ADGRG6,Akr1c19,B4GAT1,BEST1,Ca2+,CCDC85B,CPQ,CST6,FLRT1,FPR3,GAL3ST3,GPHA2,GPHB5,GPR19,GPR21,GPR85,GPR108,GPR146,GPR156,HNF1A,NPFFR2,NPS,OSM,P2RY10,PCNX3,PTH1R,SLC22A11,SLC22A12,SOX9,TGFB1,TM7SF2,TRPM5,TSHR,UGT2A3 |
| 18 | I8 | Organ Morphology, Organismal Injury and Abnormalities, Reproductive System Development and Function | 6 | 5 | BATF2,CCDC88B,Ctla2a/Ctla2b,CYP4Z1,Egfr-Erbb2,ESR2,Granzyme,IkB-NfkB,IKK (complex),IL12 (complex),IL12 (family),IL22R1-IL10R2,IRS1/2,isoleucine,Jnk,MIP1,miR-146a-5p (and other miRNAs w/seed GAGAACU),N-arachidonylglycine,OVOL1,P38 MAPK,p85 (pik3r),PI3K (complex),Pkc(s),POU6F1,Rap1-gp91-p22 phox-p40 phox-p47 phox-p67 phox,Sh2b3,SHISA2,TCR,TIGD3,TLR2/TLR4,TPST1,TRAF1-TRAF2-TRAF3,Vegf,Vegfr dimer,VWCE |
| 19 | I8 | Developmental Disorder, Hereditary Disorder, Metabolic Disease | 2 | 1 | PHLDA3,UQCC3 |
| 20 | I8 | Cancer, Organismal Injury and Abnormalities, Reproductive System Disease | 2 | 1 | SCGB1D2,SCGB2A2 |
| 21 | I8 | Hereditary Disorder, Nephrosis, Organismal Injury and Abnormalities | 2 | 1 | METTL12,NT5DC2,XPNPEP3 |
| 22 | I9 | Developmental Disorder, Hereditary Disorder, Metabolic Disease | 58 | 25 | 26sProteasome,ALK,BIRC6,BRE,caspase,CENPA,Cg,CGREF1,CYP1B1,DNMT3A,DPY30,EMILIN1,ERK1/2,FOSL2,GCKR,GPN1,HADHA,HADHB,Histone h3,Jnk,KHK,mediator,MEMO1,NFkB (complex),NLRC4,Pkc(s),PRKD3,RASGRP3,RNA polymerase II,SPAST,SPDYA,SRD5A2,SUPT7L,XDH,YWHAE |
| 23 | I9 | Auditory and Vestibular System Development and Function, Cell Death and Survival, Nervous System Development and Function | 32 | 16 | APP,ASXL2,ATRAID,CEBPZ,CIART,CLIP4,CREB1,CRIM1,DPY19L3,FBXO6,FNDC4,GIMAP8,GPR85,IGSF10,KCNC4,MT-ND5,NAGA,NDUFAF7,NMNAT2,NPM1,OCIAD2,PPM1G,PRKACA,QPCT,RAB10,RMDN2,SLC4A1AP,SLC9A6,SNX17,TMEM214,TTC27,WARS2,ZDHHC23,ZNF35,ZNF512 |
| 24 | I9 | Cellular Assembly and Organization, Cancer, Cell Morphology | 30 | 15 | 24R,25-dihydroxyvitamin D3,ABCA1,AGPAT5,ANGPT4,C3orf52,CYP4Z1,DTNB,ELAVL1,ERBB2,FAM20B,FAM98A,GALNT4,GALNT14,HEATR5B,IFT172,LBH,LCLAT1,LPCAT4,MAPK3,norepinephrine,NRBP1,NUDC,PDE7B,PREB,PRKCD,SELENOI,SLC35F6,STRN,TAS1R1,TC2N,TCF23,Vegf,VHL,WDR43,YPEL5 |
| 25 | I9 | Cell Morphology, Cellular Assembly and Organization, Nervous System Development and Function | 20 | 11 | ACTB,ADGRA1,ADGRF3,AGBL5,CDC42EP3,Clip1,DLG4,DPYSL5,EHD3,FEZ2,FOS,GPR63,GPR137,GPR146,GPR160,HNF4A,KBTBD4,KIF3C,MAPRE3,MSRB2,NGF,NUDT11,ornithine decarboxylase,PCNX1,PTEN,Ptprt,QRFPR,RBKS,SLC30A6,SORCS3,SSTR4,SSU72,TRMT61B,Uba52,VN1R1 |

| ID | Interval | Top Diseases and Functions | Score | Focus Molecules | Molecules in Network |
|---|---|---|---|---|---|
| 26 | I9 | Amino Acid Metabolism, Carbohydrate Metabolism, Molecular Transport | 18 | 10 | Akt,ANGPTL1,ARTN,BMP3,CCDC121,CD38,CLEC4A,CLEC4C,Cyp2j9,DEFB1,DRC1,EIF2B4,ERK,GAREM2,GPATCH11,IFNA2,Insulin,KDM1A,LTBP1,Na+,NANOG,NMN,NMUR2,NRG4,PLB1,PPP1CB,PRR30,S100a7a,SCD5,SLC2A9,SLC5A6,SLC8B1,SLC9A5,THEMIS2,VTCN1 |
| 27 | I9 | Cell Cycle, DNA Replication, Recombination, and Repair, Cellular Development | 2 | 1 | ABHD1,CCDC155 |
| 28 | I9 | Neurological Disease, Organismal Injury and Abnormalities, Psychological Disorders | 2 | 1 | CIB4,UBB,ZNHIT2 |
| 29 | I10 | Cell Morphology, Cellular Function and Maintenance, Cardiovascular Disease | 36 | 12 | ADAT1,ATMIN,ATP6V1C1,AZIN1,BAALC,C9orf64,CA5B,CDK5,CHEK2,CTHRC1,D-glucose,DCAF13,DZANK1,EED,ELAVL1,FZD6,GPR137,GPR180,GUF1,HCN3,HNF4A,KIAA0895,KLF10,ODF1,RIMS2,RRM2B,SLC16A5,SLC25A32,SLC43A2,SMIM7,SMIM12,TMEM101,TNF,UBR5,VN1R1 |
| 30 | I12 | Cell Cycle, Cellular Growth and Proliferation, Tissue Morphology | 22 | 8 | ADGRD1,ADGRD2,ADGRF2,ADGRF3,ADGRF4,ADGRG5,CACYBP,DEFB114,ERK1/2,Gpcr,GPR52,GPR61,GPR62,GPR82,GPR149,GPR150,GPR152,GPR157,GPR162,GPR137C,MAS1L,MRPS14,NPFFR1,PAPPA2,RABGAP1L,RB1,RFWD2,RRH,TNF,TNN,TNR,TP53,tretinoin,VN1R1,VN1R2 |
| 31 | I12 | Connective Tissue Development and Function, Connective Tissue Disorders, Nervous System Development and Function | 3 | 1 | KIAA0040,KRTAP10-3,MDFI |
| 32 | I12 | Infectious Diseases, Immunological Disease, Hematological System Development and Function | 3 | 1 | AHR,IL10RA,NXPE2 |
| 33 | I14 | Developmental Disorder, Hereditary Disorder, Immunological Disease | 26 | 10 | 5-oxo-6-8-11-14-(e,z,z,z)-eicosatetraenoic acid,ADGRG6,ALK3-BMPR2,C1q,C1QA,C1QB,C1QC,CDC42,CNR2,CR,CYP4Z1,ECE1,EPHA8,EPHB2,ERK,ERK1/2,Focal adhesion kinase,ganglioside GD2,GPR171,HSPG2,KDM1A,LAIR2,Mapk,N-arachidonylglycine,NMUR1,noladin ether,P38 MAPK,PI3K (complex),PIK3R6,Pka,RXFP3,SCARF1,STYX,TAS1R1,tetraiodothyroacetic acid |
| 34 | I14 | Cell Signaling, Nucleic Acid Metabolism, Small Molecule Biochemistry | 11 | 5 | ADCY4,ADRA2C,ADRB2,APP,BACE2,CPLX1,Dstn/Dstnl1,endocannabinoid,F2RL3,FUCA1,FZD3,GALE,GPR3,GPR6,GPR12,GPR15,GPR61,GPR78,GPR85,GPRC5B,HMGCL,HTR1B,HTR1D,LPAR3,LUZP1,MRAP2,PIK3R5,PTGDR,RXFP3,S1PR1,SCTR,SLC52A2,SNURF,SP1,VIPR2 |

| ID | Interval | Top Diseases and Functions | Score | Focus Molecules | Molecules in Network |
|---|---|---|---|---|---|
| 35 | I14 | Cellular Assembly and Organization, Developmental Disorder, Hereditary Disorder | 2 | 1 | CDC37,DLG4,FHL1,FOXB1,IER2,ZBTB40 |
| 36 | I15 | Cellular Development, Cellular Growth and Proliferation, Cell Death and Survival | 27 | 10 | BCL2,CENPC,CLOCK,DCTPP1,Defb8,EPHA5,ERMAP,HRAS,IGDCC3,IGF1,Igf1-Igfbp,IGFBP7,Il1bos,miR-1195 (miRNAs w/seed GAGUUCG),miR-153-3p (miRNAs w/seed UGCAUAG),Mucl1/Mucl2,NOA1,PCDHAC2,POLR2B,POLR2J2/POLR2J3,PPIC,RDM1,REST,RNA Pol-II-TFIIA-TFIIB-TFIID-TFIIF,RNA polymerase II,SCG3,SLC9A8,SNORD118,SPAG7,SPP1,STAT3,TMEM165,TMSB10/TMSB4X,tretinoin,ZNF74 |
| 37 | I15 | Cell Cycle, Cellular Assembly and Organization, DNA Replication, Recombination, and Repair | 6 | 3 | ADGRL3,CCDC14,CCDC18,CCDC61,CCDC77,CENPJ,CEP72,CEP120,CEP131,CEP135,CEP162,CEP290,CEP295,CEP350,CTNND1,CYLD,EXOC1,FGFR1OP,FOPNL,GYS1,KIAA0753,LUZP1,MAPRE1,MIB1,MTNR1A,NEDD1,PIBF1,PRKAR2A,PRKD1,RALA,SIPA1L1,SIPA1L2,SPATA2,TBC1D31,TMEFF1 |
| 38 | I15 | Cancer, Endocrine System Disorders, Neurological Disease | 3 | 1 | CXorf56,GOLGA1,PDCL2 |
| 39 | I16 | Cancer, Hematological Disease, Immunological Disease | 17 | 6 | ARHGAP12,beta-estradiol,Crip2,CUBN,CUL7,DNPH1,EPC1,ER-α-Estradiol,FMO1,Gm13194,GPX2,Hmgn2 (includes others),Jnk dimer,JUN,KIF5B,Magea3 (includes others),mir-383,mir-99,MYC,PADI1,PGLYRP2,Ppp1r15a,PRDM2,SCPEP1,SDK1,Snrpc,Sprr2b,Sprr2g,SVIL,TP53,TRIM6,TSKU,UBL3,YY2,ZEB1 |
| 40 | I16 | Cell Cycle, Cancer, Developmental Disorder | 3 | 1 | ERV3-1,HECW2 |
| 41 | I17 | Hair and Skin Development and Function, Cellular Compromise, Cell Morphology | 18 | 6 | ADGRG6,ANKRD42,B4GALT6,C10orf10,CCDC90B,DEFB114,DLG2,EMCN,FAM132A,FUT3,goralatide,GPR37L1,GUF1,HNF4A,MAPK3,NUDT6,NUDT11,ORM2,PAFAH2,PCF11,PFKFB4,PPP1CA,PPP1R11,PPP1R14D,PRCP,RAB30,RASGRP4,SSTR4,TMEM176B,TNF,ULBP3,UTP11,ZBTB11,ZCCHC9,ZNF300 |
| 42 | I18 | Drug Metabolism, Molecular Transport, Cellular Function and Maintenance | 28 | 11 | ADAM10,AFM,ANGPTL7,ANXA11,APP,C2orf57,CALML4,CCSER2,CDHR1,DEF6,DEFA4,DYDC1,DYDC2,ethanol,EZH2,FAM213A,Fcna,FLRT1,GIMAP6,GPR21,GRID1,KIAA0513,MAT1A,MPV17L,NME5,NRG3,OARD1,SLC13A3,TGFB1,THSD4,tretinoin,TSPAN14,TSPAN33,VAT1L,ZMIZ1 |
| 43 | I18 | Cell Morphology, Cellular Function and Maintenance, Organismal Development | 13 | 6 | ADGRD1,ADGRD2,ADGRF2,ADGRF3,ADGRF4,ADGRG5,EPO,GHITM,Gpcr,GPR27,GPR61,GPR62,GPR75,GPR82,GPR139,GPR148,GPR149,GPR150,GPR152,GPR157,GPR162,GPR137C,HNF4A,LRIT1,MAS1L,NFkB (complex),NPFFR1,OXGR1,PPIF,RGR,RRH,SFTPA1,SFTPD,VN1R1,VN1R2 |

| ID | Interval | Top Diseases and Functions | Score | Focus Molecules | Molecules in Network |
|---|---|---|---|---|---|
| 44 | I18 | Cardiovascular Disease, Hereditary Disorder, Neurological Disease | 2 | 1 | CCDC14,EMILIN1,PLAC9,RAPGEF5,RNF213 |
| 45 | I19 | Post-Translational Modification, Gene Expression, Cellular Development | 11 | 4 | Calcineurin B,CD6,DUSP2,DUSP8,DUSP16,EPHA1,EPHA4,EYA2,FARSB,FZD10,G0S2,glycochenodeoxycholate,GSTA2,GSTM2,HNF4α dimer,IL36B,Jnk,mir-101,MKP2/5,NUDT1,PAX3,PPP1R16B,PPP2R5B,PRAM1,PTPRH,RLBP1,S1PR4,S1PR5,SGPP2,SH2D3C,sphingosine-1-phosphate,sphingosine-1-phosphate phosphatase,SRGN,TFF2,tretinoin |
| 46 | I19 | Cell-mediated Immune Response, Cellular Movement, Hematological System Development and Function | 3 | 1 | 2-acylglycerol O-acyltransferase,AGPAT2,BSCL2,Ccl2,CD44,CXCL10,diacylglycerol O-acyltransferase,MOGAT1,TIMP1,triacylglycerol |

**Table S9.** Transcription factor binding sites for the *PPARG* gene.

| ID | Target Name | Interval | *Cis/Trans*-eQTL | Associated Gene |
|---|---|---|---|---|
| 1 | ABHD1 | I9 | *trans* | *FABP4* |
| 2 | ADIPOQ | | | |
| 3 | AGPAT2 | | | |
| 4 | AHNAK | I8 | *trans* | *FABP4* |
| 5 | ALK | I9 | *trans* | *FABP4* |
| 6 | ATL3 | I8 | *trans* | *FABP4* |
| 7 | ATP6V1C1 | I10 | *trans* | *FABP4* |
| 8 | AZIN1 | I10 | *trans* | *FABP4* |
| 9 | B3GNT2 | I3 | *trans* | *ACSM5* |
| 10 | BAD | I8 | *trans* | *FABP4* |
| 11 | BATF2 | I8 | *trans* | *FABP4* |
| 12 | BEST1 | I8 | *trans* | *FABP4* |
| 13 | C1QA | I14 | *trans* | *FADS2* |
| 14 | C2orf16 | I9 | *trans* | *FABP4* |
| 15 | C2orf71 | I9 | *trans* | *FABP4* |
| 16 | CAPN14 | I9 | *trans* | *FABP4* |
| 17 | CD248 | I8 | *trans* | *FABP4* |
| 18 | CD36 | | | |
| 19 | CDC42BPG | I8 | *trans* | *FABP4* |
| 20 | CDC42EP2 | I8 | *trans* | *FABP4* |
| 21 | CDC42EP3 | I9 | *trans* | *FABP4* |
| 22 | CDH6 | I6 | *trans* | *ACSM5* |
| 23 | CDHR1 | I18 | *trans* | *SLC27A4* |
| 24 | CENPA | I9 | *trans* | *FABP4* |
| 25 | CEP135 | I15 | *trans* | *FADS2* |
| 26 | CHRM1 | I8 | *trans* | *FABP4* |
| 27 | CIB4 | I9 | *trans* | *FABP4* |
| 28 | CLOCK | I15 | *trans* | *FADS2* |
| 29 | CPSF7 | I8 | *trans* | *FABP4* |
| 30 | CPT1A | | | |
| 31 | CRIM1 | I9 | *trans* | *FABP4* |
| 32 | CST6 | I8 | *trans* | *FABP4* |
| 33 | CTHRC1 | I10 | *trans* | *FABP4* |
| 34 | CTXN3 | I1 | *trans* | *ACSM5* |
| 35 | CUBN | I16 | *trans* | *FADS2* |
| 36 | CYP2U1 | | | |
| 37 | DAGLA | I8 | *trans* | *FABP4* |
| 38 | DDB1 | I8 | *trans* | *FABP4* |
| 39 | DGAT1 | | | |
| 40 | DGAT2 | | | |

| ID | Target Name | Interval | *Cis/Trans*-eQTL | Associated Gene |
|---|---|---|---|---|
| 41 | *DLG2* | I17 | *trans* | *SLC27A4* |
| 42 | *DNMT3A* | I9 | *trans* | *FABP4* |
| 43 | *ECE1* | I14 | *trans* | *FADS2* |
| 44 | *EHBP1* | I3 | *trans* | *ACSM5* |
| 45 | *EHD3* | I9 | *trans* | *FABP4* |
| 46 | *ELOVL5* | | | |
| 47 | *EMILIN1* | I9 | *trans* | *FABP4* |
| 48 | *EML3* | I8 | *trans* | *FABP4* |
| 49 | *EPAS1* | I4 | *trans* | *ACSM5* |
| 50 | *EPC1* | I16 | *trans* | *FADS2* |
| 51 | *EPHA8* | I14 | *trans* | *FADS2* |
| 52 | *EPT1* | I9 | *trans* | *FABP4* |
| 53 | *ESRRA* | I8 | *trans* | *FABP4* |
| 54 | *FABP4* | | | |
| 55 | *FADS1* | I8 | *trans* | *FABP4* |
| 56 | *FADS2* | I8.I13 | *trans/cis* | *FABP4/FADS2* |
| 57 | *FADS3* | I8 | *trans* | *FABP4* |
| 58 | *FAM179A* | I9 | *trans* | *FABP4* |
| 59 | *FKBP2* | I8 | *trans* | *FABP4* |
| 60 | *FLRT1* | I8 | *trans* | *FABP4* |
| 61 | *FNDC4* | I9 | *trans* | *FABP4* |
| 62 | *FOSL1* | I8 | *trans* | *FABP4* |
| 63 | *FOSL2* | I9 | *trans* | *FABP4* |
| 64 | *FRMD8* | I8 | *trans* | *FABP4* |
| 65 | *GAL3ST3* | I8 | *trans* | *FABP4* |
| 66 | *GALNT14* | I9 | *trans* | *FABP4* |
| 67 | *GCKR* | I9 | *trans* | *FABP4* |
| 68 | *GGA2* | I5 | *trans* | *ACSM5* |
| 69 | *GPR52* | I12 | *trans* | *FABP4* |
| 70 | *GRID1* | I18 | *trans* | *SLC27A4* |
| 71 | *HADHA* | I9 | *trans* | *FABP4* |
| 72 | *HSPG2* | I14 | *trans* | *FADS2* |
| 73 | *IGFBP7* | I15 | *trans* | *FADS2* |
| 74 | *KCNH8* | I7 | *trans* | *ELOVL6* |
| 75 | *KHK* | I9 | *trans* | *FABP4* |
| 76 | *KIAA0040* | I12 | *trans* | *FABP4* |
| 77 | *KIF5B* | I16 | *trans* | *FADS2* |
| 78 | *KLC2* | I8 | *trans* | *FABP4* |
| 79 | *KLF10* | I10 | *trans* | *FABP4* |
| 80 | *LBH* | I9 | *trans* | *FABP4* |

| ID | Target Name | Interval | *Cis/Trans*-eQTL | Associated Gene |
|---|---|---|---|---|
| 81 | *LCLAT1* | I9 | *trans* | *FABP4* |
| 82 | *LIPC* | | | |
| 83 | *LMNB1* | I1 | *trans* | *ACSM5* |
| 84 | *LPIN1* | | | |
| 85 | *LRRC10B* | I8 | *trans* | *FABP4* |
| 86 | *LTBP1* | I9 | *trans* | *FABP4* |
| 87 | *LTBP3* | I8 | *trans* | *FABP4* |
| 88 | *LUZP1* | I14 | *trans* | *FADS2* |
| 89 | *MACROD1* | I8 | *trans* | *FABP4* |
| 90 | *MAP4K2* | I8 | *trans* | *FABP4* |
| 91 | *MAPRE3* | I9 | *trans* | *FABP4* |
| 92 | *MARCH3* | I1 | *trans* | *ACSM5* |
| 93 | *MARK2* | I8 | *trans* | *FABP4* |
| 94 | *MDH1* | I3 | *trans* | *ACSM5* |
| 95 | *MEGF10* | I1 | *trans* | *ACSM5* |
| 96 | *MGLL* | | | |
| 97 | *MIR194-2* | I8 | *trans* | *FABP4* |
| 98 | *MLXIPL* | | | |
| 99 | *MOGAT1* | I19 | *trans* | *SLC27A4* |
| 100 | *MRPL49* | I8 | *trans* | *FABP4* |
| 101 | *NR1H3* | | | |
| 102 | *NRXN2* | I8 | *trans* | *FABP4* |
| 103 | *NUDT22* | I8 | *trans* | *FABP4* |
| 104 | *OVOL1* | I8 | *trans* | *FABP4* |
| 105 | *PAPPA2* | I12 | *trans* | *FABP4* |
| 106 | *PCNXL3* | I8 | *trans* | *FABP4* |
| 107 | *PDZD2* | I6 | *trans* | *ACSM5* |
| 108 | *PEX2* | | | |
| 109 | *PLA2G16* | I8 | *trans* | *FABP4* |
| 110 | *PLAC9* | I18 | *trans* | *SLC27A4* |
| 111 | *PLCB2* | | | |
| 112 | *PLCB3* | I8 | *trans* | *FABP4* |
| 113 | *PNPLA2* | | | |
| 114 | *POU2F1* | | | |
| 115 | *PPARA* | | | |
| 116 | *PPARG* | | | |
| 117 | *PPIF* | I18 | *trans* | *SLC27A4* |
| 118 | *PPM1B* | I4 | *trans* | *ACSM5* |
| 119 | *PPP1R14B* | I8 | *trans* | *FABP4* |
| 120 | *PRKCE* | I4 | *trans* | *ACSM5* |

| ID | Target Name | Interval | *Cis/Trans*-eQTL | Associated Gene |
|---|---|---|---|---|
| 121 | *PRKD3* | I9 | *trans* | *FABP4* |
| 122 | *PRRC1* | I1 | *trans* | *ACSM5* |
| 123 | *PYGM* | I8 | *trans* | *FABP4* |
| 124 | *RAB30* | I17 | *trans* | *SLC27A4* |
| 125 | *RAB3IL1* | I8 | *trans* | *FABP4* |
| 126 | *RABGAP1L* | I12 | *trans* | *FABP4* |
| 127 | *RASGRP2* | I8 | *trans* | *FABP4* |
| 128 | *RASGRP3* | I9 | *trans* | *FABP4* |
| 129 | *RBP4* | | | |
| 130 | *RCOR2* | I8 | *trans* | *FABP4* |
| 131 | *REST* | I15 | *trans* | *FADS2* |
| 132 | *RHOQ* | I4 | *trans* | *ACSM5* |
| 133 | *RIMS2* | I10 | *trans* | *FABP4* |
| 134 | *RTN3* | I8 | *trans* | *FABP4* |
| 135 | *RXRG* | | | |
| 136 | *SATB1* | I7 | *trans* | *ELOVL6* |
| 137 | *SCD* | | | |
| 138 | *SF3B2* | I8 | *trans* | *FABP4* |
| 139 | *SH2D4B* | I18 | *trans* | *SLC27A4* |
| 140 | *SLC22A11* | I8 | *trans* | *FABP4* |
| 141 | *SLC22A12* | I8 | *trans* | *FABP4* |
| 142 | *SLC25A45* | I8 | *trans* | *FABP4* |
| 143 | *SLC27A1* | | | |
| 144 | *SLC27A4* | | | |
| 145 | *SLC5A6* | I9 | *trans* | *FABP4* |
| 146 | *SNX15* | I8 | *trans* | *FABP4* |
| 147 | *SNX17* | I9 | *trans* | *FABP4* |
| 148 | *SREBF1* | | | |
| 149 | *STRN* | I9 | *trans* | *FABP4* |
| 150 | *SYT7* | I8 | *trans* | *FABP4* |
| 151 | *TAF6L* | I8 | *trans* | *FABP4* |
| 152 | *TCF23* | I9 | *trans* | *FABP4* |
| 153 | *TMEM138* | I8 | *trans* | *FABP4* |
| 154 | *TMEM165* | I15 | *trans* | *FADS2* |
| 155 | *TNN* | I12 | *trans* | *FABP4* |
| 156 | *TNR* | I12 | *trans* | *FABP4* |
| 157 | *TRPT1* | I8 | *trans* | *FABP4* |
| 158 | *TSPAN14* | I18 | *trans* | *SLC27A4* |
| 159 | *TUT1* | I8 | *trans* | *FABP4* |
| 160 | *UBR5* | I10 | *trans* | *FABP4* |

| ID | Target Name | Interval | *Cis/Trans*-eQTL | Associated Gene |
|---|---|---|---|---|
| 161 | *VIT* | I9 | *trans* | *FABP4* |
| 162 | *VWCE* | I8 | *trans* | *FABP4* |
| 163 | *XDH* | I9 | *trans* | *FABP4* |
| 164 | *ZBTB40* | I14 | *trans* | *FADS2* |
| 165 | *ZEB1* | I16 | *trans* | *FADS2* |
| 166 | *ZMIZ1* | I18 | *trans* | *SLC27A4* |
| 167 | *ZNF438* | I16 | *trans* | *FADS2* |

**Table S10.** Pearson's correlations of *PPARG* mRNA expression and analyze-related genes.

| Gene | Pearson correlation coefficient | *p*-value |
|---|---|---|
| ***ACSM5*** | 0.08 | $3.86x10^{-1}$ |
| ***ADIPOQ*** | 0.66 | $1.78x10^{-15}$ |
| ***AGPAT2*** | 0.54 | $6.98x10^{-10}$ |
| ***ANK2*** | 0.27 | $3.93x10^{-3}$ |
| ***ARNT*** | 0.30 | $1.42x10^{-3}$ |
| ***CD36*** | 0.70 | $2.22x10^{-16}$ |
| ***CPT1A*** | 0.03 | $7.33x10^{-1}$ |
| ***CROT*** | 0.50 | $1.71x10^{-8}$ |
| ***CYP2U1*** | 0.54 | $6.01x10^{-10}$ |
| ***DGAT1*** | 0.62 | $2.53x10^{-13}$ |
| ***DGAT2*** | 0.61 | $1.09x10^{-12}$ |
| ***EGF*** | 0.08 | $4.00x10^{-1}$ |
| ***ELOVL5*** | 0.75 | $2.22x10^{-16}$ |
| ***ELOVL6*** | 0.68 | $2.22x10^{-16}$ |
| ***ESRRA*** | 0.43 | $2.41x10^{-6}$ |
| ***FABP4*** | 0.51 | $9.27x10^{-9}$ |
| ***FABP5*** | 0.40 | $1.27x10^{-5}$ |
| ***FADS1*** | 0.12 | $1.95x10^{-1}$ |
| ***FADS2*** | 0.18 | $5.92x10^{-2}$ |
| ***FADS3*** | 0.17 | $7.71x10^{-2}$ |
| ***LIPC*** | 0.27 | $3.55x10^{-3}$ |
| ***LPIN1*** | 0.69 | $2.22x10^{-16}$ |
| ***MGLL*** | 0.43 | $1.90x10^{-6}$ |
| ***MLXIPL*** | 0.61 | $5.55x10^{-13}$ |
| ***NFKB1*** | 0.58 | $2.49x10^{-11}$ |
| ***NR1H3*** | 0.56 | $1.56x10^{-10}$ |
| ***PEX2*** | 0.60 | $1.69x10^{-12}$ |
| ***PLA2G12A*** | 0.61 | $9.13x10^{-13}$ |
| ***PLCB2*** | 0.13 | $1.71x10^{-1}$ |
| ***PLPP1*** | 0.27 | $4.16x10^{-3}$ |
| ***PNPLA2*** | 0.55 | $3.09x10^{-10}$ |
| ***POU2F1*** | 0.25 | $7.75x10^{-3}$ |
| ***PPARA*** | 0.63 | $1.01x10^{-13}$ |
| ***PPARD*** | 0.48 | $8.61x10^{-8}$ |
| ***RBP4*** | 0.36 | $1.11x10^{-4}$ |
| ***RXRG*** | 0.41 | $7.83x10^{-6}$ |
| ***SCAP*** | 0.45 | $4.59x10^{-7}$ |
| ***SCD*** | 0.59 | $1.01x10^{-11}$ |
| ***SLC27A1*** | 0.23 | $1.23x10^{-2}$ |
| ***SLC27A4*** | 0.23 | $1.60x10^{-2}$ |
| ***SREBF1*** | 0.55 | $2.63x10^{-10}$ |
| ***USF1*** | 0.33 | $4.15x10^{-4}$ |

**Table S11.** Primers used for the characterization of the 3'UTR of porcine *FABP4* gene.

| Gene | Full name | Primer Name | Type | Primer Sequence[1] |
|---|---|---|---|---|
| *FABP4* | *Fatty acid binding protein 4* | oligo(dT)-UAP | mRNA 3' UTR amplification | 5'-GGCCACGCGTCGACTAGTAC(T)30VN-3' |
| *FABP4* | *Fatty acid binding protein 4* | UAP | mRNA 3' UTR amplification | 5'-GGCCACGCGTCGACTAGTAC-3' |
| *FABP4* | *Fatty acid binding protein 4* | FABP4-3NC-1-Fw | mRNA 3' UTR sequencing | 5'-TTGGATCGAACTCTACAACACTCTG-3' |
| *FABP4* | *Fatty acid binding protein 4* | FABP4-3NC-2-Fw | mRNA 3' UTR sequencing | 5'-TGTTTATGGATCTTCCATTATCTTAGG-3' |

[1]Primers were designed from the GenBank Y16039 sequence

**Table S12.** Primers used for the analyses of gene expression of the 48 genes by real-time PCR.

| Gene | Full name | Type | Primer sequence | |
|---|---|---|---|---|
| | | | Forward | Reverse |
| *ACSM5* | Acyl-CoA synthetase medium-chain family member 5 | Target | 5'-TGTAATCTGTGCCAATCCCAAA-3' | 5'-CATCATCTACGATCTGCACCTCAT-3' |
| *ACTB* | Actin, Beta | Reference | 5'-CAAGGACCTCTACGCCAACAC-3' | 5'-TGGAGGCGCGATGATCTT-3' |
| *ADIPOQ* | Adiponectin, C1Q and collagen domain containing | Target | 5'-GTACCCCAGGCCGTGATG-3’ | 5’- CCCTTAGGACCAGTAAGACCTGTATCT-[illegible] |
| *AGPAT2* | 1-acylglycerol-3-phosphate O-acyltransferase 2 | Target | 5’-CATGGTCAGGGAGAAGCTCAA-3’ | 5’-GCCAGGTAGAAGGCACCTTTC-3’ |
| *ANK2* | Ankyrin 2 | Target | 5'-GTGGATTCTGCTACGAAGAAAGG-3’ | 5'-AAGGACTTTGACAACTTCTGCTTGT-3’ |
| *ARNT* | Aryl hydrocarbon receptor nuclear translocator | Target | 5’-TCTAATGATAAGGAGCGGTTTGC-3’ | 5’-TATGATTTTCCCTGGCGAGTCT-3’ |
| *B2M* | Beta-2-microglobulin | Reference | 5’- ACCTTCTGGTCCACACTGAGTTC-3’ | 5’- GGTCTCGATCCCACTTAACTATCTTG-3[illegible] |
| *CD36* | CD36 molecule | Target | 5'-GGTCCTTACACGTACAGAGTTCGTT-3’ | 5'-CCATTGGGCTGTAGGAAAGAGA-3’ |
| *CPT1A* | Carnitine palmitoyltransferase 1A | Target | 5’-CCTGAAGGTGCTGCTCTCCTA-3’ | 5’-CTCACCATCATCATCCAGATCTTG-3’ |
| *CROT* | Carnitine O-octanoyltransferase | Target | 5'-GGGAAACGAAATTGGTTGGA-3' | 5'-CGCAAAGTTGACATTCAGTTGTG-3' |
| *CYP2U1* | Cytochrome P450, family 2, subfamily U, member 1 | Target | 5’-AGAGAAAACAGTGCTCCAAGGGTAT-3’ | 5’-TGGCTGGGTCTCTGTGTACTGA-3’ |
| *DGAT1* | Diacylglycerol O-acyltransferase 1 | Target | 5'-CCTGAATTGGTGTGTGGTCATG-3' | 5'-GATGCCGTACTTGATGAGGTTCTC-3' |
| *DGAT2* | Diacylglycerol O-acyltransferase 2 | Target | 5'-GGAACACGCCCAAGAAAGGT-3’ | 5'-GGATGGGAAAGTAGTCTCGAAAGTAG-3[illegible] |
| *EGF* | Epidermal growth factor | Target | 5’-AACGGGAATGCCACTTGTGT-3’ | 5’-CCTTCCAAGTCAATCCTAAAGATACTG-3[illegible] |
| *ELOVL5* | ELOVL fatty acid elongase 5 | Target | 5’-CCTCTCGGCTGGCTGTACTT-3’ | 5’-CCTTCTTGTTGTAGGTCTGGATGTAG-3[illegible] |
| *ELOVL6* | ELOVL fatty acid elongase 6 | Target | 5’-AGCAGTTCAACGAGAACGAAGCC-3’ | 5’-TGCCGACCGCCAAAGATAAAG-3’ |
| *ESRRA* | Estrogen-related receptor alpha | Target | 5’-CAAGAGCATCCCAGGCTTCTC-3’ | 5’-CACCCAACACCAATACCTCCAT-3’ |
| *FABP4* | Fatty acid binding protein 4 | Target | 5'-TAAGTTGGTGGTGGAATGTATCATG-3' | 5'-AGAGTGTTGTAGAGTTCGATCCAAAC-3[illegible] |
| *FABP5* | Fatty acid binding protein 5 | Target | 5'-CCAATGGAGAATTGGTTCAACA-3' | 5'-GTTCATGACGCATACCACCACTA-3' |
| *FADS1* | Fatty acid desaturase 1 | Target | 5’-CCTTGTGAGGAAGTATATGAGCTCTCT-3’ | 5’-TCATCTGTCAGCTCTTTATTCTTAGTCG-[illegible] |
| *FADS2* | Fatty acid desaturase 2 | Target | 5’-TCCACCGCGACCTTGATTTA-3’ | 5’-TCGGTGATCTCAGAGTTCTTGGT-3’ |
| *FADS3* | Fatty acid desaturase 3 | Target | 5’-CCAGCACCTCTACTTCTTCCTGAT-3’ | 5’-CATGTATGCCAGATTTTCCACTTC-3’ |

| Gene | Full name | Type | Primer sequence | |
|---|---|---|---|---|
| | | | Forward | Reverse |
| *HPRT1* | Hypoxanthine phosphoribosyltransferase 1 | Reference | 5’- TCATTATGCCGAGGATTTGGA-3’ | 5’- CTCTTTCATCACATCTCGAGCAA-3’ |
| *LIPC* | Lipase C, hepatic type | Target | 5’- CGTTACAGCAGTGTGGCTTCA-3’ | 5’- CCAGCAAGCCATCCATCAA-3’ |
| *LPIN1* | Lipin 1 | Target | 5’- CCGAGAGAAGGTGGTGGACAT-3’ | 5’- CTCTCCATTGTCTCCCAGTTTCA-3’ |
| *MGLL* | Monoglyceride lipase | Target | 5'-GTGTTCGCCCACGACCAT-3’ | 5'-CCTGACGAACACCTGGAAGTC-3’ |
| *MLXIPL* | MLX Interacting Protein-Like | Target | 5’-CCCAAGTGGAAGAATTTCAAAGG-3’ | 5’-CTTCCTCCGCTCCACATACTG-3’ |
| *NFKB1* | Nuclear factor kappa B subunit 1 | Target | 5'-CCCACAGACGTTCATAGACAATTT-3' | 5'-GAGGCTGGTTTTGTAATGTTGACA-3' |
| *NR1H3* | Nuclear Receptor Subfamily 1, Group H, Member 3 | Target | 5'-CTGGGCATGATCGAGAAGCT-3' | 5'-TGGGCCAAGGCGTGACT-3' |
| *PEX2* | Peroxisomal biogenesis factor 2 | Target | 5'-CTCAGACTCCTAAGAAACCTTCAGAGA-3' | 5'-ACTGATTCTGAGCACTCTGTTTGC-3' |
| *PLA2G12A* | Phospholipase A2, group XIIA | Target | 5'-CCCACTCTTTGGTGTTCATCTTAA-3' | 5'-ATAGCACCTGTCGTGCTGGTT-3' |
| *PLCB2* | Phospholipase C, beta 2 | Target | 5’- AGATCTTCACTTCCTACCACTCCAA-3’ | 5’- GCGCTGTTTCTGGTTGATGA-3’ |
| *PLPP1* | Phospholipid phosphatase 1 | Target | 5'-GGCCACTCTTCATTCTCCATGTAC-3' | 5'-AGGCCCACGTAAATGGATACAG-3' |
| *PNPLA2* | Patatin-like phospholipase domain containing 2 | Target | 5'-CTTCACCGTCCGCTTGCT-3’ | 5'-GCATCACCAGGTACTGGCAGAT-3’ |
| *POU2F1* | POU class 2 homeobox 1 | Target | 5’- CCCATACAGATCGCACAGGAT-3’ | 5’- GATGATAAACTGTGCTGGTTGCA-3’ |
| *PPARA* | Peroxisome proliferator-activated receptor alpha | Target | 5'-GGCACTGAACATCGAATGTAGAATC-3' | 5'-CCGAAAGAAGCCCTTGCAA-3' |
| *PPARD* | Peroxisome proliferator-activated receptor delta | Target | 5'-GCATGTCTCA CAACGCCATT-3' | 5'-GCTGACTCCCCTCGTTTGC-3' |
| *PPARG* | Peroxisome proliferator-activated receptor gamma | Target | 5'-TTGTGAAGGATGCAAGGGTTT-3’ | 5'-ATCCGACAGTTAAGATCGCACCTA-3’ |
| *PPARGC1A* | Peroxisome proliferator-activated receptor gamma, coactivator 1 alpha | Target | 5’-CTCTGGAACTGCAGGCCTAA-3’ | 5’-TGGAGAAGCCCTAAAGGGTTAT-3’ |
| *RBP4* | Retinol binding protein 4 | Target | 5'-GGGTCGAGTCCGTCTTTTAAATAACT-3’ | 5'-GGTCCTCGGTGTCTGTAAAGGT-3’ |
| *RXRG* | Retinoid X receptor, gamma | Target | 5'-GAGGATTCTGGAAGCTGAACTTG-3' | 5'-TCATTCGTCGAATTCTCCATGT-3' |
| *SCAP* | SREBF chaperone | Target | 5’- AGATATCTCAGGCCTTCTACAACCA-3’ | 5’- AGTTTCAACAGTGGGTAGCAGC-3’ |
| *SCD* | Stearoyl-CoA Desaturase | Target | 5’- GGTGATGTTCCAGAGGAGGTACTAC-3’ | 5’- CAGCAATACCAGGGCACGAT-3’ |
| *SLC27A1* | Solute carrier family 27, member 1 | Target | 5'-TCACTCGGCAGGGAACATC-3’ | 5'-CGGCTGGCTGAAAACTTCTT-3’ |
| *SLC27A4* | Solute carrier family 27, member 4 | Target | 5'-GCTGCATAAAACAGGGACTTTCA-3’ | 5'-AACAGCGGGTCTTTCACGACT-3’ |

| Gene | Full name | Type | Primer sequence | |
|---|---|---|---|---|
| | | | Forward | Reverse |
| *SREBF1* | Sterol Regulatory Element Binding Transcription Factor 1 | Target | 5'-CACGGAGGCGAAGCTGAATA-3' | 5'-GCTTCTGGTTGCTCTGCTGAA-3' |
| *TBP* | TATA-Box Binding Protein | Reference | 5'-CAGAATGATCAAACCGAGAATTGT-3' | 5'-CTGCTCTGACTTTAGCACCTGTTAA-3' |
| *USF1* | Upstream transcription factor 1 | Target | 5’- CCCTTATTCCCCGAAGTCAGA-3’ | 5’- GCGGCGTTCCACTTCATTAT-3’ |
